# Enhancing healthcare at home for older people in rural and regional Australia: A protocol for co-creation to design and implement system change

**DOI:** 10.1371/journal.pone.0290386

**Published:** 2023-09-08

**Authors:** Cindy Needham, Nikita Wheaton, Anna Wong Shee, Kevin McNamara, Mary Malakellis, Margaret Murray, Laura Alston, Anna Peeters, Anna Ugalde, Catherine Huggins, Serene Yoong, Steven Allender

**Affiliations:** 1 Institute for Health Transformation, Global Centre for Preventative Health and Nutrition, School of Health and Social Development Faculty of Health, Deakin University, Geelong, Australia; 2 Deakin Rural Health, School of Medicine, Faculty of Health, Deakin University, Geelong, Australia; 3 Community and Aged Care, Grampians Health, Ballarat, Victoria, Australia; 4 Research Unit, Colac Area Health, Colac, Victoria, Australia; 5 Institute for Health Transformation, Faculty of Health, Deakin University, Geelong, Australia; UNITED KINGDOM

## Abstract

**Background:**

World-wide, health service providers are moving towards innovative models of clinical home-based care services as a key strategy to improve equity of access and quality of care. To optimise existing and new clinical home-based care programs, evidence informed approaches are needed that consider the complexity of the health care system across different contexts.

**Methods:**

We present a protocol for working with health services and their partners to perform rapid identification, prioritisation, and co-design of content-appropriate strategies to optimise the delivery of healthcare at home for older people in rural and regional areas. The protocol combines Systems Thinking and Implementation Science using a Consensus Mapping and Co-design (CMC) process delivered over five workshops.

**Discussion:**

The protocol will be implemented with rural and regional healthcare providers to identify digital and non-digital solutions that have the potential to inform models of service delivery, improve patient experience, and optimise health outcomes. The combination of system and implementation science is a unique approach for optimising healthcare at home for older populations, especially in the rural context where need is high. This is the first protocol to integrate the use of systems and implementation science into one process and articulating these methods will help with replicating this in future practice. Results of the design phase will translate into practice through standard health service planning methods to enhance implementation and sustainability. The delivery of the protocol will include building capacity of health service workers to embed the design, implementation, and evaluation approach into normal practice. This protocol forms part of the DELIVER (Delivering Enhanced heaLthcare at home through optImising Virtual tools for oldEr people in Rural and regional Australia) Project. Funded by Australia’s Medical Research Future Fund, DELIVER involves a collaboration with public health services of Western Victoria, Australia.

## Background

Ageing populations present one of the most significant health care challenges of this century, and novel approaches are needed to ensure health systems can cope with the increasing burden of age-related disease or ill-health [1]. Reducing hospital admissions for older adults is a key focus of governments and health services globally, to better manage healthcare outcomes and the associated costs [2–4]. Potentially avoidable hospital admissions for older Australians cost 1.9 million patient days and $3.7 billion in 2020–21 [5]. The burden increases with age and the geographical remoteness of the patient [6]. More than half of these preventable admissions (61%) relate to chronic conditions, many of which may be avoided through timely, quality primary care, and management at home [6–9]. Home-based care [10] involves two key strategies 1) the delivery of acute or subacute admitted services in a person’s home and 2) acute health services, working with community and social services to provide community-based, non-admitted care or health care services [11, 12]. To date home-based care has been underutilised, due to difficulties addressing the needs for an often heterogenous and complex patient and dependence on face-to-face interactions [11, 12]. Challenges with providing and accessing appropriate home-based care are exacerbated in marginalised populations; in for example, rural, regional, and remote communities [13]. Any attempt to address these challenges needs to take into consideration the contextual drivers of these challenges and the relevant evidence to ensure feasibility and fit [14]. To optimise engagement and ownership, best practice suggests to optimise home-based care programs and/or to address the modifiable drivers of this complex problem, requires a multi-level and integrated approach developed in partnership with stakeholders for which the prevention would be directed [14].

The World Health Organization (WHO) recommends that system science be used to strengthen the implementation of evidence-based practice in health services [15]. Evidence-based practice is defined as the integration of clinical experience, knowledge, and patient values, with best research evidence in the decision-making process for care provision [16, 17]. The WHO further describes building blocks that form the ‘health system’ 1) health services 2) health workforce 3) health information 4) equitable access to essential medical products, vaccines and technologies 5) health financing 6) leadership and governance [15]. Taken together, these elements represent a complex interplay of factors that are likely to differ by health service and context. System science offers unique methods to understand the relationships between all the parts and players within a system and describe outcomes that are valuable to the health service (i.e., intended, and unintended consequences, effects of actions and timing and delay between actions and outcomes) [18, 19]. Group Model Building (GMB) is a recommended method for using system science [20], through a series of facilitated workshops, key stakeholders explore the causes and consequences of complex problems and generate potential interventions informed by current evidence [20, 21]. GMB is more effective than traditional facilitation methods at generating tailored and contextually appropriate solutions through stakeholder consensus, given its user-friendly participatory approach which supports individual and collective changes in group-decision contexts [22, 23].

The use of implementation science, to translate evidence into routine practice, has been suggested as a another key approach that could be utilised in addressing health inequities, such as those experienced by rural and regional Australians [24]. The Implementation Science Framework EPIS (Exploration, Preparation, Implementation and Sustainment) [21, 25] has been used to inform multi-component implementation strategies in the public service sector [26]. However, Implementation Scientists highlight the complex challenges of selecting the most appropriate implementation strategy given the large array of discrete implementation strategies described in published taxonomies [27–29]. Powell et al suggests that GMB may be one effective method to guide the process for selecting and tailoring implementation strategies to address the unique needs of implementation methods [27]. We suggest that the combination of Implementation Science in turn with GMB could be complementary and has the potential to build the capacity of health services to effectively implement the actions identified [16, 17]. This is important because poor implementation is often cited as one of the main reasons that actions generated as part of GMBs do not achieve the desired outcomes [26]. Despite this, a clear protocol outlining how the two approaches of system and implementation science can be used in tandem to identify the most appropriate interventions and their implementation represents a significant gap in knowledge. We propose to test the hypothesis that utilising systems and implementation science approaches will be an effective and acceptable process for health services to identify and plan for the implementation of appropriate interventions to optimise home-based care for older adults in regional and rural areas. This protocol describes in detail the pre-work required to prepare for co-creation events. This is rarely described in detail and is often left to chance or driven by team members with implementation experience.

### Aim

The aim of this paper is to present a protocol for applying systems science and implementation science approaches to working with health services, their partners and consumer representatives to perform rapid identification, prioritisation, and co-design of strategies to optimise the delivery of clinical care services at home or closer to home for older people in rural and regional Australia.

## Methods

### Design

GMB based on community based participatory system dynamics and implementation science using the EPIS framework. GMB is a participatory approach which involves engaging diverse stakeholders in workshops to gain a joint understanding of a complex issue and develop a consensus for action [23]. EPIS was selected as this framework is flexible to the dynamic relationship between the outer and inner contexts and considers that adaptation is probable over all phases of implementation [30].

### Setting

This study is set within the region of Western Victoria, Australia and will engage multiple health services classified using the Modified Monash Model (MMM) as MMM 2–5 (i.e., large regional centres and large, medium and small rural towns) and one health service recently classified as MMM1 [31].

### Stakeholders

Stakeholders will be involved in the planning, preparation and facilitation of workshops, including members of the DELIVER Research Team (henceforth Research Team), which includes embedded health service researchers, academics with expertise in systems and implementation science and consumer representatives. Within each stage, identification of participants to engage with will be guided by the National Implementation Research Network (NIRN) guidelines which highlights the importance of having participants involved that understand the population, the problem, the community and that are empowered to engage in the decision-making [32, 33].

1. An **Organisational liaison** will act as a ‘boundary spanner’ and act as a bridge between health services and the research team. Organisational liaisons will be identified by leadership from each health service to assist the Research Team with data collection, initiative inventory and identifying and recruiting participants for workshops guided by the NIRN Guidance for Engaging Critical Perspectives [32, 33].
2. **Workshop participants** will be adults over the age of 18 years who fit one (or more) of the following three categories; ***Consumers or Consumer representatives***: older people in rural Australia who may be eligible for healthcare in the home, their carers and family; and people with lived experience, or representatives of people with lived experience of the health system. ***Organisational leaders***: Health service organisational leaders with high level strategic understanding of health service and regulatory body policies and procedures. ***Organisational stakeholders***: Operational and service delivery staff (clinical and non-clinical) of health services and other organisations engaged in healthcare in the home with lived experience of the organisational system.

### Procedure summary—Consensus Mapping and Co-design (CMC)

**Fig 1:**
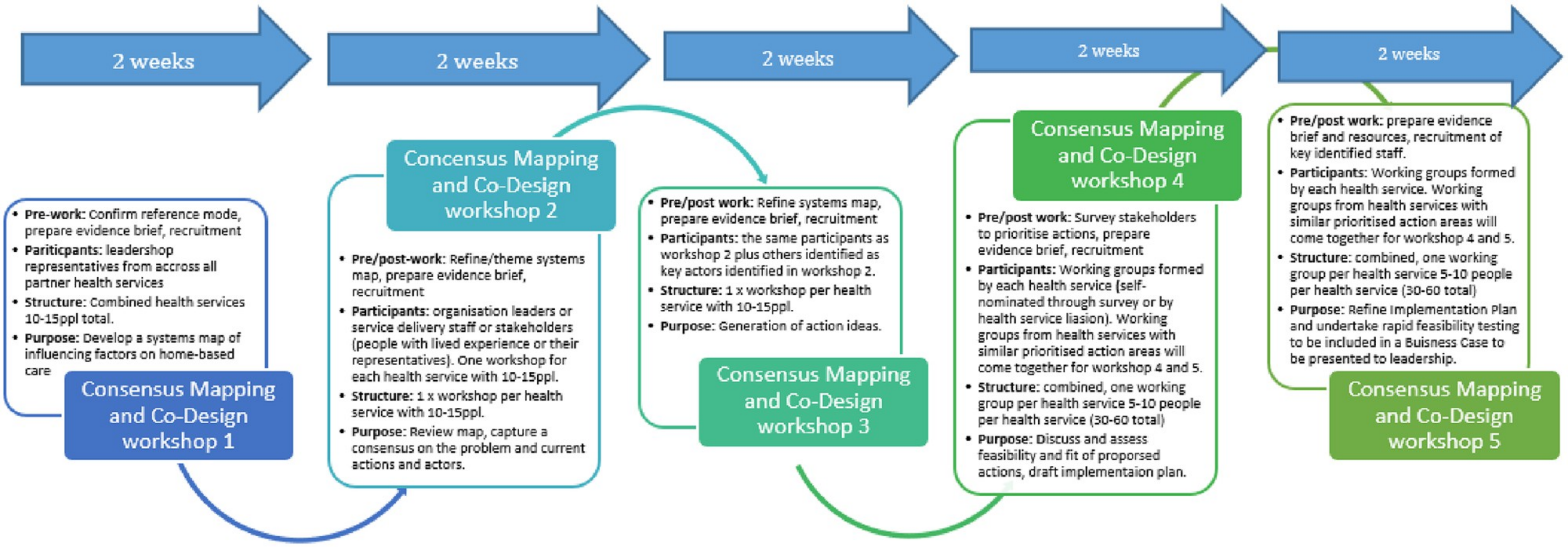
Structure for DELIVER consensus mapping and Co-design for local solutions.

The five CMC workshop process aims to understand the system of healthcare delivered at home-or closer to home for older people and identify and prioritise local actions and potential for new innovations guided by evidence-based practice (described in section 1–5 below). The results are stakeholder led co-created action plans which can be presented to the organisational leadership team with the aim of seeking endorsement, resourcing, implementation and evaluation support. Fig 1 and Table 1 summarise the objectives, outcomes and anticipated participants for each CMC workshop. This protocol (Part A-E) describes in detail the workshop-related activities required before, during and after each workshop. Table 2 summarises the tools and frameworks used throughout. Each CMC will utilise Implementation Science tools and frameworks and will inform the basis for the Exploration and Preparation phase of the EPIS Framework [25, 30, 34].

**Table 1:** Consensus Mapping and Co-design (CMC) process.

Session	EPIS stage	Objective	Participants	Outcome
1	Exploration	Engage key stakeholders and create initial CLD of the problem.	Combined representatives for health services across the region: organisational leads & stakeholders/consumers or consumer representatives.	Overall causal loop diagram representing the system; and the factors that influence the problem within the system, and how they interact. Provides insight into common themes and feedback loops associated with the problem.
2	Exploration	Review causal loop diagram which has been themed by the research team; and capture existing actions.	Health service level: organisational leads & stakeholders/consumers or consumer representatives; key people identified in CMC workshop 1.	Engage a broader group of stakeholders from each individual health service and surrounding community to contribute to the CLD built in the CMC1 for their local health service. The CLD is then used as a basis to identify any key actors and actions already active or occurring within the system; where and what action should be strengthened, reduced, or new solutions created.
3	Exploration	Translate map insights into action ideas.	Health service level: organisational staff and stakeholders/consumers, others identified in CMC workshop 2.	Validation of the causal loop diagram and themes. Identify current actions, and areas where actions are needed. Propose broad action areas.
4	Preparation	Assess feasibility and fit of prioritised actions and draft Implementation Plan.	Combined representatives for health services across the region: organisational staff with influence/responsibility for areas where actions have been prioritised and developed.	Develop specific actions areas that may positively influence the problem, identify key stakeholders linked to actions. Convene working groups for each health service around each prioritised Action Area; within working groups organisations working together to assess contextual fit and feasibility of actions guided by guided by NIRN’s Hexagon tool [35]; and commence Implementation Planning for the most highly ranked action guided by the P-O-L-C Framework, a strategic management framework that emphasises the essential components needed in planning to enable an action (or vision) to be successfully implemented [36, 37].
5	Preparation	Undertake rapid feasibility testing; explore opportunities for human/financial implementation support; Implementation Plan refinement; and development of Business Case.	Combined representatives for health services across the region: Working groups from each health service including organisational staff with influence/ responsibility for areas where actions have been prioritised and developed.	Implementation strategy developed and tested by simulation within the system. Implementation Plan for action at the organisation level prepared and ready for presentation to leadership. Capacity building of organisational working group participants to capture and track actions against initial system map, feedback across the system to optimise interventions

**Table 2:** Frameworks and tools used throughout the Consensus Mapping and Co-design process.

Framework/Tool	Description	Scientific tool/approach	
Exploration, Preparation, Implementation and Sustainment (EPIS) Framework	The Exploration, Preparation, Implementation and Sustainment (EPIS) framework (Fig 1) is a four phased guiding framework for the identification and implementation of new program or practice [26]. The framework was developed through summation of the implementation literature in the public sectors of social and allied health in the USA [26].	Implementation Science	The EPIS Framework will be used to inform the integration of systems science and implementation science approaches
Causal Loop Diagram (CLD)	A CLD is a visual tool used in systems science for conceptualising a problem or issue and understanding the feedback loops between factors within the system [38].	Systems Science	A visual tool for mapping factors that influence a complex problem and how they interact.
Hexagon Tool	The Hexagon tool is a guide for discussion and analysis for organisations to evaluate new and existing programs and practices through discussing and rating six domains which examine and rate the contextual fit and feasibility of an action, program or practice (Evidence, Usability, Supports, Capacity to Implement, Fit with Current Initiatives, Need) [35].	Implementation Science	The ratings for each domain are between 1 (least supported by the discussion) to five (most supported). The Evidence briefs presented to stakeholders during the workshops will inform discussion for the ‘Evidence’ domain under the Program Indicators; and the CLD will be used to represent the Need indicators under the Implementing Site Indicators. Each of the questions in the remaining domains under the Program Indicators (Usability and Supports) and Implementing Site Indicators (Capacity to Implement and Fit with Current Initiatives) will be considered with input from participants [35].
Systems Thinking for Community Knowledge Exchange (STICKE) Software [39]	STICKE software creates a visual representation (CLD) of the group’s shared understanding of the problem. STICKE also allows practitioners to theme and group factors, track where action is already happening; where future efforts should be focused; assess feasibility and capacity to deliver actions; and track actions within the system as they evolve.	Systems Science	Software utilised throughout workshops to develop causal loop diagrams.
Planning Organising Leading and Controlling (P-O-L-C)	Planning Organising Leading and Controlling (P-O-L-C) Framework which breaks down the four functions that need to be considered when planning to achieve an objective within an organisation [36, 37].	Strategic Management	The POLC frameworks supports participants to develop a 1) Plan comprising objectives and strategies, locating and confirming 2) Organisational functions (financial, operational or human), identify and engage 3) Leadership through considering leaders motivations 4) Control relating to setting performance standards and monitoring [36, 37].

Ethical approval for the Consensus Mapping and Co-Design process outlined in this protocol has been provided following review by the Barwon Health Human Research Ethics (HREC) Committee (ID: 94753) and the approved protocol submitted for consideration to the HREC has been submitted with the manuscript as a S1 File.

Each of the five face-to-face CMC Workshops (CMC-1–CMC-5) will run for approximately three hours and will be delivered by trained facilitators using scripted activities [40] informed by preparatory and summary activities conducted before and after each session (Tables 3–6) [40]. Pre- and post- workshop activities will take approximately one week each side of the workshop with the entire process ideally delivered over a 2-month period to ensure continued momentum of the project and information retention. Due to the implications of COVID-19 and potential geographic constraints all workshops will have the potential to be delivered online and over a longer period.

**Table 3:** Workshop format and data collection for session one (Consensus Mapping and Co-design workshop 1).

Agenda item	Time (mins)	Description
Welcome	*10*	The study lead introduces the session and the purpose of the study, welcomes people to the session and outlines the meeting structure and aims.
Evidence Brief	*20*	Participants are presented with an evidence brief providing the most recent evidence regarding the topic area (problem statement) and data evidencing the state of play at the regional level. The evidence brief also presents information on what is known about implementing interventions and/or programs within the context of the topic area.
Behaviours over time	*20*	Participants are presented with the reference mode comprising the change over time at the organisational and regional level of the identified problem. Participants are then asked to consider, individually, what factors are leading to, or resulting from, the patterns observed in the reference mode. Participants are asked to express these as a function of how the factor has changed (behaviour) over time. Participants then work in small groups of three to determine which of these factors should be prioritised in describing the cause and effect of the problem at the regional level.
Connection circle	*45*	Each of the small working groups present their priority variables in turn and these are entered into the STICKE software. STICKE collects these variables and participants are then asked to identify the relationship of cause and effect between any of the variables collected. For each relationship (for e.g., digital literacy and access to healthcare advice) participants consider the most likely relationship of cause and effect, and the direction of the relationship (i.e., how does change in one variable affect change in another). The STICKE software then translates these causal relationships into an overall logic model showing each of the variables and their connections.
Model review	*15*	Participants review the initial model and confirm they are happy with the contents and relationships or add and edit as they see fit.
Session Close	*05*	

**Table 4:** Workshop format and data collection for session two (Consensus Mapping and Co-design workshop 2).

Agenda item	Time (mins)	Description
**Welcome**	*10*	The study lead introduces the session and the purpose of the study, welcomes people to the session and outlines the meeting structure and aims.
**Evidence Brief**	*15*	Participants are presented with the revised evidence brief providing the most recent information about topic area (problem statement). The evidence brief will provide more detailed information about interventions and existing local actions relating to the CLD collected in step one.
**Model review introduction**	*15*	The process used to develop the CLD in STICKE is described to the participants. The map is presented in stages to participants by building the map up theme by theme. The meaning of the variables, direction and style of arrows representing the causal relationships are described to participants.
**Model Discussion/Workshop**	*25*	Participants work in small groups of 2–3 people to read through their map on large printed copies (minimum A3 size) of the map and consider their health service specific home-based care programs. Participants are asked to consider factors that are missing from the map that should be included, consider any clarifications, talk about feedback loops, and give general feedback.
**Model Update**	*25*	Participants share their most important change/s or update to the map. The Modeller captures important changes in STICKE as they are described and notes are taken summarising the discussion in the room.
**Break**	*15*	
**Mini Action Ideas and Workshop 3 Prep**	*40*	Participants are introduced to Action Ideas and that the third workshop will be about inviting a larger group of people to identify and plan action. Participants working in small groups are invited to review the CLD of the system and place a black dot where they feel there is something important, and a pink dot where they feel something is missing. The facilitator encourages participants to discuss potential actions in light of those considerations and to consider the other key groups or organisations in the community who ought to be part of a detailed conversation around action at the next workshop. Participants are encouraged to make notes to leave for the facilitation team, or to email suggestions to a nominated contact.
**Group summary to room**	*15*	The small working groups created are invited to share 1–2 ideas. attendees to consider the other key groups or organisations in the community who ought to be part of a detailed conversation around action at the next workshop. Participants are again encouraged to make notes to leave for the facilitation team, or to email suggestions to a nominated contact
**Next steps and close**	*5*	The next steps in the project are described and the meeting drawn to a close.

**Table 5:** Workshop format and data collection for session three (Consensus Mapping and Co-design workshop 3).

Agenda item	Time (mins)	Description
Welcome	*10*	The study lead introduces the session and the purpose of the study, welcomes people to the session and outlines the meeting structure and aims.
Evidence Brief	*15*	Participants are presented with the revised evidence brief providing the most recent information about topic area (reference mode) in the context of the particular health service.
**Action Capture**	*15*	Participants are asked to brainstorm what is already happening to address *the problem* in their particular health service and community. These actions are captured on paper listing 1) the name of the activity 2) organisations involved 3) brief description of activity.
**Model Presentation**	*10*	The map is presented in stages to participants by building the map up theme by theme. The meaning of the variables, direction and style of arrows representing the causal relationships are described to participants.
**Model Action Area Prioritization**	*10*	Using these augmented maps participants to; identify the places on the map where existing action is happening and describe this along with the part of the map the action would affect; consider where more action is needed and describe this action and the place on the map they feel it would act; and highlight areas of the map where they feel they have power and agency to act. These responses will be recorded on handouts and will identify existing actions, areas for more action and the potential (power to act) at the local organisation level.
Action ideas introduction and generating	*20*	Participants are asked to brainstorm action ideas and record them on action idea templates first working individually for the next 5 minutes then in small groups for a further 15 minutes. Identifying action ideas. The process used to develop the CLD and action registers in STICKE is described to the participants actions areas from step two are introduced.
Prioritise	*15*	Working in small groups of 2 or 3 people, participants are then asked to share their ideas with each other and prioritise these ideas in order from highest to lowest priority. They are asked to prioritise considering both the feasibility of the action and the likely impact of the action. And informed by their existing knowledge and experience.
Group summary to room	*20*	The small working groups created in the previous step report their priority actions to the rest of the group and these actions are recorded and displayed at the front of the room.
Working group formation	*30*	Participants are asked to choose a working group based on the prioritised action area that best meets their current remit, interest and capacity. The working group then reviews the actions in their thematic area, prioritising these in order of likely impact and feasibility. The team then conducts its first working group meeting, collecting the names of the participants, their preferred level of commitment to the group, identifying other stakeholders that may need to be involved relevant to their local health service. Participants will also consider the actions through the lens of the WHO Health Systems Strengthening framework [15] and consider what success would look like and what resources would be required.
Group summary to room	*20*	The small working groups created in the previous step report their priority actions to the rest of the group and these actions are recorded and displayed at the front of the room. Participants given the opportunity for participants to contribute and provide feedback to other working groups.
Next steps and close	*5*	The next steps in the project are described and the meeting drawn to a close.

**Table 6:** Workshop format and data collection for step four (Consensus Mapping and Co-design workshop 4).

Agenda item	Time (mins)	Description
Welcome	*10*	The study lead introduces the session and the purpose of the study, welcomes people to the session and outlines the meeting structure and aims.
Evidence Brief	*10*	Participants are presented with the revised evidence brief providing the most recent evidence of the effectiveness of prioritised action area and actions, the availability of evidence-based programs or guidance for best practice.
Hexagon tool introduction	*25*	Participants are introduced to the Hexagon discussion and analysis tool and the questions that sit under each domain, and rating sheets [35].
Working group formation—Hexagon Tool	*30*	Participants are asked to work in small groups with representatives from their organisation. Each group will be asked to systematically work through the Hexagon tool considering the Evidence, Supports, Usability, Need, Fit and Capacity to Implement of up to three specific actions prioritised within the Action Area [35]. Each indicator will then be ranked to reflect the feasibility and fit of each action considered with discussion points noted.
Group summary to room	*20*	Each group will report their ratings from the Hexagon tool for their specific actions that were discussed and analysed and other subgroups will be given the opportunity to provide comment and discuss.
Working group—Setting a Business Case: Implementation Planning	*30*	Each group will be asked to draft an Implementation Plan for the most feasible and fitting action (i.e., most highly ranked) for their organisation sequentially using each of the four functions outlined in the P-O-L-C Framework [37] 1) Planning 2) Organising 3) Leading 4) Controlling.
Group summary to room	*20*	Each subgroup will briefly present the Implementation Plan and identify the next steps to be taken in the next 0–3 months.
Next steps and close	*5*	The next steps in the project are described and the meeting drawn to a close.

### CMC 1: Engage and develop initial casual loop diagram of the identified problem at the regional level

#### Objective

To develop a consensus map of the system that illustrates the factors that broadly drive access to clinical home-based care and their feedback relationships. A secondary objective is to build the capacity of participants to use systems thinking to understand the complex system in which they operate.

#### Participants and recruitment

The Organisational Liaisons from each participating health service will assist with identifying, engaging and recruiting key organizational leaders or stakeholders to participate in CMC1. It is anticipated that there will be 10–15 participants from multiple health services. Participants will include organisational leaders, who have local and/or broader (i.e., state or country) understandings of the challenges facing the organisation or consumers.

Participants will be invited via email by the Organisational Liaison to attend the series of five workshops and sent a Participation Information and Consent Form for review. The participation Information and Consent Form will be provided in hard copy to all participants (and new participants in subsequent workshops) at the commencement of each workshop. It is expected as the participants and engagement narrow to the organisational/local level, many of these stakeholders may opt to delegate local responses to staff within each organisation. Each participant will receive a briefing document (flyer) which will provide an overview of the CMC process to improve their understanding of the systems science approach and the CMC process and their role before commencing CMC1.

### Pre-workshop activities

1. Confirm (joint) problem: Working with the Organisational Liaisons and leadership, the research team will confirm the specific aspect of home-based care to be addressed. Described in system science as a **Reference Mode**, the problem statement describes the outcome of interest and the way in which it has changed over time. For example, reference modes might include changing rates of digital health literacy among older adults or admissions that were potentially avoidable through home-based care. Semi-scripted interview questions tailored to the organisation will be used to confirm the reference mode with the organisational leader.
2. Conduct rapid review: A rapid review will be conducted for the reference mode, current evidence will be identified and synthesised describing current patterns relating to the problem and the current evidence about effective solutions [41]. The review will provide an overview of implementation and service outcomes that have influenced the Reference Mode (e.g., interventions that have influenced rates of potentially avoidable hospital admissions). Organisational data will also be collected to inform the problem statement and assist with evaluation (i.e., baseline measures) as well as to identify key organisation representatives and stakeholders.
3. The results of the rapid review and the organisational data collected will be combined to form the basis of an evidence brief which will be presented to participants in the first CMC workshop. The evidence brief will be up to 20 minutes in length and tailored to ensure it is understandable to all participants.
4. Recruit and organise CMC1: Participants for CMC1 will be organisation leaders who have a regional or broader understanding of the challenges facing the organisations. They will be asked to identify others who fit this criterion and have not yet been contacted. It is anticipated that the number of participants will be between 10 and 15 (two or more from each health service).
  Step 2 (Rapid Review) and Step 3 (Recruitment) will likely occur concurrently.

### Workshop activities

The method underpinning the workshop approach is community-based participatory system dynamics [20], which comprises a suite of tightly scripted activities to identify relationships of cause and effect relating to the problem statement. The activities are facilitated by the Research Team and notes are taken during the process to capture the depth of discussion to inform later refinement and review. Table 2 provides an overview of workshop format and data collection for CMC2. It should be noted that the workshop format where necessary can be adapted to meet the logistical needs of delivery (e.g., delivered online or over two sessions), however the activities should cover the key activities outlined.

### Post workshop activities

#### Workshop outcome

This step will build a causal loop diagram (CLD) using STICKE software (Table 3) based on stakeholders’ understanding of local context and the evidence of the factors driving the reference mode at the regional level. This will provide a visual understanding of the system in which the reference mode is operating, the factors that are influential and how they interact. This will align outcomes with the exploration phase within EPIS [30].

### CMC 2: Capture a consensus on the problem and current actions and actors within the context of each health service—Where are current actions, where are actions needed, where can we act?

#### Objective

To engage a broader group of stakeholders from each individual health service and surrounding community including consumers or consumer representatives, organisational leaders, and organisational stakeholders, to contribute to the CLD built in the CMC1 for their local health service. The CLD is then used as a basis to identify key actors and actions already active or occurring within the system; where and what action should be strengthened, reduced, or new solutions created.

### Pre-workshop activities

1. CLD Review and Evidence brief review: Following CMC1 the Research Team will review and refine the CLD based on workshop notes and identify emerging thematic areas. The evidence brief from CMC1 will be revised to include the variables not covered in the first evidence brief. An inventory of relevant existing initiatives will be collected to provide a clear picture of existing programs or initiatives, polices, strategies and resource commitments in place for each health service [42]. These materials will then be used as a part of CMC2. **Note**: Where key stakeholders are unable to attend the CMC2 a condensed one-on-one session (online or in-person) will be conducted with the stakeholder (up to two per organisation) led by the Organisational Liaison with feedback provided to the Research Team were relevant.
2. Recruit and organise: CMC2 will work with individual health services, the same participants that attended CMC1 will be invited to the second session along with additional health service and consumer representatives. It is anticipated that 10–15 people will take part in each health service specific workshop. The NIRN Guidance [43] will be used to assess and plan for the engagement of a diverse range of perspectives relevant to the topic area in consultation with the Organisational Liaison.

### Workshop activities

Table 4 provides an overview of workshop format and data collection for CMC2.

### Post workshop activities

#### Outcome

The CLD will be further developed in the context for each individual health service reflecting their specific programs and/or initiatives. The updated model will be confirmed as accurate from the participants point of view for their health service (i.e., each health service will have a refined CLD that reflects the context of their health service). A clear list of existing actions and actors will be identified, and participants will identify priority areas for strengthening existing action or creating new action in the system. Participants are introduced to Action Ideas and spend time generating action ideas.

### CMC 3: Generating action ideas and create working groups

#### Objective

To translate the results of the previous two sessions and generate action ideas; and form working groups around specific action areas/ideas.

### Pre-workshop activities

1. CLD and Evidence brief review: Following CMC2 the Research Team will review and refine the CLDs to include existing and proposed actions and actors. The Research Team will use workshop notes to refine the CLD, complete the inventory of current initiatives, and confirm stakeholders. The map will be themed by types of action proposed and working groups formed for these themes. **Working Groups (WG)** will function beyond the workshop series described here to develop and implement actions generated throughout the process. Evidence briefs will be extended to ensure coverage of prioritised action areas.
2. Recruit and organise: CMC3 will be run for each health service individually and participants will comprise organisational leaders and stakeholders identified by participants in CMC1-2. This will involve 10 to 15 stakeholders (fewer for smaller health services) per health service leading to engagement with between 100 and 150 participants across the region.

### Workshop activities

Table 5 provides an overview of workshop format and data collection for CMC3.

### Post workshop activities

#### Outcomes

This workshop will create a list of actions (and the actors identified as working on them/responsible for their delivery areas) based on perceived feasibility and likely impact. Working groups will have been formed around broad action areas and actions clearly defined and considered in terms of existing resources and support.

### CMC 4: Feasibility and fit of actions and co-design of an implementation plan

#### Objective

To consolidate working groups across organisations based on their shared priority action areas and confirm the feasibility and fit of the proposed actions. Co-design of an implementation plan and business case for each agreed action.

### Pre-workshop activities

1. CLD and Evidence brief review: Following CMC3 the Research Team will review the CLD to create sub maps relevant to each of the workshop action areas. Prior to the workshop, the Initiative Inventory will be checked for accuracy with stakeholders via email. A rapid review of the evidence for prioritised action areas will be undertaken to assess the potential effectiveness of prioritised action areas and actions in addressing the problem. Grey literature along with other relevant databases such as the Effective Practice and Organisation of Care (EPOC) reviews [44] and Cochrane Handbook for Systematic Reviews of Interventions [45] will also be searched for supporting evidence on best-practice.
2. Action prioritisation: Action areas (e.g., digital literacy) and actions (e.g., education program to increase digital literacy) defined in step three will be prioritised for each individual health service via the Qualtrics survey platform. The survey will be sent to the participants so far engaged in each CMC and snowballed to a wider audience of stakeholders to confirm the top three priority action areas and actions for each health service; Participants will also be given the opportunity to self-nominate which working groups they would like to join.
3. Recruit and organise: CMC3 Participants will be invited to join CMC4 which will form the basis for establishing health service networks (i.e., grouping health services that intend to deliver a similar action, allowing for collaboration and information sharing). One workshop will be held for each of the top three (or more) prioritised action areas and will be attended by multiple health services who also prioritised the same action area.

### Workshop activities

Table 6 provides an overview of workshop format and data collection for CMC4. At commencement of the workshops, health service specific working groups will be established. The Hexagon discussion and analysis tool [35] questions will be used to discuss, analyse and rank the feasibility and fit of up to three actions with participants. Each working group will be given a handout (or electronic copy) of the Hexagon tool on which they will record the numerical rating for each of the six indicators; and take notes to record their discussion and rating justification. Actions that have been positively assessed for feasibility and fit will then be operationalised using the P-O-L-C Framework [37].

### Post workshop activities

#### Outcomes

An implementation plan, comprising the most feasible and fitting prioritised actions under each of the P-O-L-C functions. A regional strategy document comprising implementation plans for each working group will be compiled including localised adaptations for each individual organisation (Table 6). A network of organisations seeking to implement similar interventions will have been formed.

### CMC 5: Implementation planning refinement, feasibility testing and business case preparation

#### Objective

To refine a context specific Implementation Plan for each health service, build capacity to use systems and implementation science approaches, undertake simulated feasibility testing and prepare a business case for organisational leaders or funding bodies. Create a network across health services in the region for knowledge exchange and communication based on chosen actions.

#### Procedure (Pre-work)

1. Recruit and organise: Participants will be those who have committed to a particular ‘action’ working group in CMC4; further stakeholders identified as required to ‘support’ implementation will be invited to join the action working group.

### Workshop activities

CMC5 sets up and describes the routine operation of the health service working groups based on the most feasible and fitting actions identified in CMC4. The working group will finalise implementation plans for their health service, identifying required resources, capacity, infrastructure, training, timelines, data collection and evaluation. The working group will use STICKE with assistance from the Research Team to add the new action into the CLD and assess the feasibility that the action will influence intended targets within the system; identify potential unintended consequences and identify other new factors that may affect implementation. Working groups will capture changes through their business case and create and routinely update an action register to track process changes.

### Post workshop activities

#### Outcomes

A completed implementation plan for the delivery of an action within their organisation. Implementation plans will be able to inform a business case that includes a feasibility test, that can be presented to leadership to support requests for support, resourcing and future implementation. Participants will also have gained the skills required to track and assess the success and unintended consequences of the action within the CLD at a later stage of implementation. It is recommended that further co-design will involve engagement with consumers to test actions to validate the effectiveness of the actions planned for implementations.

### Outcome measures and analysis

Several specialised systems analysis methods may be applied to these data across the group of communities to assess their reach and effectiveness. Specific analyses will include 1) Network analysis, which can be applied to one or more of the systems maps that have arisen from the GMB process from one or more participating health services to identify structurally important features within the systems map. This can be used to help identify points of similarity or difference/uniqueness between communities, or between comparable maps informed by literature or expert opinion. 2) Implementation and action tracking over time: CLDs may be aligned with information about the status and progress of actions over time and be compared across health services and will create a real-time resource for monitoring the implementation of actions from a systems-thinking perspective 3) Case study/reflective qualitative analyses: Case studies afford an opportunity to qualitatively analyse insights from the range of potential data sources. These analyses will focus on reflective, practitioner focussed, applied research which may present studies on successful implementation of systems practice in health services, tracing insights from GMB, through to the actions informed by those insights (tracked through implementation records) and concordant implications for community outcomes and implementation practise (through outcome and systems practise evaluations). The broader DELIVER project will include additional evaluation into the future to measure the increase in translation of research for health care at home service delivery for older people; improvements in health services and health professional’s confidence to deliver and support home-based care using digital and non-digital tools; and, whether more people are able to access home-based care or closer to home services confidently.

## Discussion

This protocol provides a detailed description of how system and implementation science could complement each other and support co-creation of locally led solutions to complex health problems. The consensus mapping co-design process combines the participatory approach of GMB to understand and identify the complex system informing a topic area/problem; and intersects this with implementation science, using tools to identify the most feasible and fitting intervention (action) to create positive improvements within the organisation. The process will lead to the creation of an evidence informed business case or strategy, assessed for both feasibility and fit and located within existing organisational systems.

The protocol responds to the gap identified by the WHO in presenting the guide for health system strengthening, in which they call for system and implementation science to be utilised together when operationalising the framework for action [14]. Additional components to the GMB process, such as those presented in this protocol, are needed to ensure the substantial resources, time investment and high-level buy-in required by an organisation to take part in the process is more likely to have a positive return on investment [20, 21, 23]. By expanding the traditional GMB format to include additional components supported by implementation science frameworks and tools, the protocol presents a process whereby actions generated at completion of the process may be more actionable; and the likelihood of actions being implemented is improved.

### Strengths

Implementation of this protocol is supported by a large, funded program with strong engagement from health services with an established need to address the challenges of delivering clinical services at home or closer to home in regional and rural areas. DELIVER involves a partnership with eight public health services and collaboration with several smaller health services across the western region of Victoria, Australia; and an operational team that is comprised of researchers and health service representatives with a diversity of skills who will support the process at different stages. Systems thinking provides a way to operate more successfully in complex, real-world settings and resolve health system challenges in a way that is more context appropriate and allows for the tackling of areas for improvement within complex health systems [14]. A systems thinking approach allows us to better understand the complexity of a health system and design interventions that will optimize health and health equity [14]. The inclusive participatory approach used in systems science has the added benefit of establishing ownership of the processes and outcomes within the system [14]. By incorporating systems methods and implementation science tools and frameworks, this protocol extends upon the traditional GMB process and supports the integration of evidence-based practice which is highly rigorous in an engaging format. Utilising tools such as the Hexagon tool will assist in the selection of a suitable approach(es) for intervention/action, and that the implementation context is considered prior to approaching implementation [35]. The description of these steps is a strength of this protocol as it supports replicability of the approach and therefore makes steps towards a generalisable method. In addition, the use of STICKE software to create a complex visual map which can assist in simulation modelling of an action, be reflected on in future and be used to track action progress is invaluable.

### Limitations

Whilst we have incorporated all efforts to include the voice of all stakeholders and actors in the system throughout the CMC process, there are logistical challenges when bringing together diverse players given the complex power imbalances that may exist across organisations. To overcome this, we have included an opportunity for individual consultation and a survey to engage a broader audience within the process of action idea prioritisation and working group formation, however we acknowledge that these efforts may not suffice in engaging hard to reach stakeholders.

The delivery of this protocol relies on significant human resources to undertake the evidence synthesis, recruitment and facilitation of workshops which may be out of scope for some organisations. However, we propose that investment in this comprehensive process which intersects systems science with implementation science is more likely to have a positive and long-lasting impact compared to utilising the approaches individually. The goal of this protocol is to present a process for combining both Systems and Implementation Science and if successful then we would look at where efficiencies could be found in future iterations.

### Implications

This protocol will be implemented with rural and regional public healthcare providers to identify digital and non-digital solutions to embed into home-based clinical care to improve service delivery, patient experience, and health outcomes for the communities they serve and provide an opportunity to evaluate this approach.

Through DELIVER, the Research Team, in partnership with health services and stakeholders will develop co-designed local solutions to improve clinical healthcare services for older persons at home or closer to home across the western region of Victoria, Australia. The application and evaluation of this approach will be reported in future research with the intention that it can be applied in other settings. Prioritised actions identified through the CMC workshops will move into the second stage of the DELIVER process (the Implementation Phase) which will involve further co-design and trialling of the actions.

## Conclusion

Applying systems science and implementation approaches to overcome complex issues within organisations have the potential to overcome the challenges faced through utilising these approaches independently. The approaches complement each other and provide a clear process to identify the most appropriate intervention in a complex system that requires supporting evidence for implementation to occur.

## Supporting information

S1 File (DOCX)

## Abbreviations

Action Area: Broad action area i.e., alternate ED platforms
Action: intervention, specific to what will be done i.e., digital 24hour ED platform
CLD: Causal Loop Diagram
CMC: Consensus Mapping and Co-Design
EPIS Framework: Exploration Preparation Implementation and Sustainment Framework
GMB: Group Model Building
Hexagon tool: a tool designed to help organizations evaluate new and existing programs and practices. This tool is designed to be used by a team to ensure diverse perspectives are represented in a discussion of the six contextual fit and feasibility factors [35]
Working Group: working groups will be established around Action Areas and will be comprised of self-nominated (or nominated) representatives from multiple organisations that have prioritised that Action Area and Actions within it for their organisation

## References

[1] WHO. Ageing and health Online 2022 [https://www.who.int/news-room/fact-sheets/detail/ageing-and-health.

[2] Huntley AL, Chalder M, Shaw ARG, Hollingworth W, Metcalfe C, Benger JR, et al. A systematic review to identify and assess the effectiveness of alternatives for people over the age of 65 who are at risk of potentially avoidable hospital admission. BMJ open. 2017;7(7):e016236. doi: 10.1136/bmjopen-2017-016236 28765132 PMC5642761

[3] D’Souza S, Guptha S. Preventing admission of older people to hospital. BMJ (Clinical research ed). 2013;346:f3186. doi: 10.1136/bmj.f3186 23690461

[4] van den Broek S, Heiwegen N, Verhofstad M, Akkermans R, van Westerop L, Schoon Y, et al. Preventable emergency admissions of older adults: an observational mixed-method study of rates, associative factors and underlying causes in two Dutch hospitals. BMJ open. 2020;10(11):e040431. doi: 10.1136/bmjopen-2020-040431 33444202 PMC7682455

[5] AMA. Putting health care back into aged care. Online: https://www.ama.com.au/articles/report-putting-health-care-back-aged-care-0.

[6] Falster MJ, L.,. A guide to the potentially preventable hospitalisations indicator in Australia. Sydney: University of New South Wales in consultation with Australian Commission on Safety and Quality in Health Care and Australian Institute of Health and Welfare; 2017.

[7] McManamny TE, Boyd L, Sheen J, Lowthian JA. Health initiatives to reduce the potentially preventable hospitalisation of older people in rural and regional Australia. Health Promotion Journal of Australia. 2022;33(3):553–65. 34494699 10.1002/hpja.539

[8] Clinical Epidemiology and Health Service Evaluation Unit. Potentially preventable hospitalisations: a review of the literature and Australian policies. Melbourne, Victoria: Royal Melbourne Hospital; 2009.

[9] Australian Institue of Health and Welfare. Disparities in potentially preventable hospitalisations across Australia, 2012–13 to 2017–18. Canberra: AIHW; 2020.

[10] Commonwealth of Australia. Royal Commission into Aged Care quality and Safety. Prime Minister and Cabinet; 2021.

[11] Victorian Clinical Council. Home-based care becoming the new norm. 2020.

[12] Healthcare Improvement Scotland. Hospital at Home: Guiding principles for service development. 2020.

[13] AIHW. Rural and Remote Health Online: https://www.aihw.gov.au/reports/rural-remote-australians/rural-and-remote-health: Australian Government; 2022.

[14] WHO. Systems thinking for health systems strengthening. Geneva: World Health Organisation; 2009.

[15] WHO. Strenthening health systems to improve health outcomes: WHO’s framework for action. Geneva: World Health Organisation; 2007.

[16] Sackett D. Evidence based medicine: how to practice and teach EBM. Second edition. ed: Churchill Livingstone; 2000.

[17] Grimshaw JM, Eccles MP, Lavis JN, Hill SJ, Squires JE. Knowledge translation of research findings. Implementation Science. 2012;7(1):50–66. doi: 10.1186/1748-5908-7-50 22651257 PMC3462671

[18] Allender S, Owen B, Kuhlberg J, Lowe J, Nagorcka-Smith P, Whelan J, et al. A Community Based Systems Diagram of Obesity Causes. PLoS ONE. 2015;10(7):1–12. doi: 10.1371/journal.pone.0129683 26153893 PMC4496094

[19] Mabry PL, Olster DH, Morgan GD, Abrams DB. Interdisciplinarity and systems science to improve population health: a view from the NIH Office of Behavioral and Social Sciences Research. American Journal of Preventive Medicine. 2008;35(2(Suppl. 1)):S211–S24. doi: 10.1016/j.amepre.2008.05.018 18619402 PMC2587290

[20] Hovmand PS. Community based system dynamics. New York, NY: Springer Science + Business Media; 2014.

[21] Gerritsen S, Harré S, Rees D, Renker-Darby A, Bartos AE, Waterlander WE, et al. Community Group Model Building as a Method for Engaging Participants and Mobilising Action in Public Health. International journal of environmental research and public health. 2020;17(10). doi: 10.3390/ijerph17103457 32429183 PMC7277214

[22] Scott RJ, Cavana RY, Cameron D. Recent Evidence on the Effectiveness of Group Model Building. European Journal of Operational Research. 2016;249(3):908–18.

[23] Siokou C, Morgan R, Shiell A. Group model building: a participatory approach to understanding and acting on systems. Public health research & practice. 2014;25(1). doi: 10.17061/phrp2511404 25828443

[24] Kerkhoff AD, Farrand E, Marquez C, Cattamanchi A, Handley MA. Addressing health disparities through implementation science-a need to integrate an equity lens from the outset. BioMed Central; 2022. p. 1–4. 35101088 10.1186/s13012-022-01189-5 PMC8802460

[25] Becan JE, Bartkowski JP, Knight DK, Wiley TRA, DiClemente R, Ducharme L, et al. A model for rigorously applying the Exploration, Preparation, Implementation, Sustainment (EPIS) framework in the design and measurement of a large scale collaborative multi-site study. Health & justice. 2018;6(1):9. 29654518 10.1186/s40352-018-0068-3 PMC5899075

[26] Aarons GA, Hurlburt M, Horwitz SM. Advancing a conceptual model of evidence-based practice implementation in public service sectors. Administration and Policy in Mental Health and Mental Health Services Research. 2011;38(1):4–23. doi: 10.1007/s10488-010-0327-7 21197565 PMC3025110

[27] Powell BJ, Beidas RS, Lewis CC, Aarons GA, McMillen JC, Proctor EK, et al. Methods to Improve the Selection and Tailoring of Implementation Strategies. The journal of behavioral health services & research. 2017;44(2):177–94. doi: 10.1007/s11414-015-9475-6 26289563 PMC4761530

[28] Powell BJ, McMillen JC, Proctor EK, Carpenter CR, Griffey RT, Bunger AC, et al. A compilation of strategies for implementing clinical innovations in health and mental health. Medical Care Research and Review. 2012;69(2):123–57. doi: 10.1177/1077558711430690 22203646 PMC3524416

[29] Powell BJ, Waltz TJ, Chinman MJ, Damschroder LJ, Smith JL, Matthieu MM, et al. A refined compilation of implementation strategies: results from the Expert Recommendations for Implementing Change (ERIC) project. Implementation Science. 2015;10(1):1–14. doi: 10.1186/s13012-015-0209-1 25889199 PMC4328074

[30] Moullin J, Dickson K, Stadnick N, Rabin B, Aarons G. Systematic review of the Exploration, Preparation, Implementation, Sustainment (EPIS) framework. Implementation Science. 2019;14(1):1–16. doi: 10.1186/s13012-018-0842-6 30611302 PMC6321673

[31] Department of Health and Aged Care. Modified Monash Model 2022 [https://www.health.gov.au/health-topics/rural-health-workforce/classifications/mmm.

[32] National Implementation Research Network. Guidance for Engaging Critical Perspectives 2023.

[33] International Association for Public Participation. Spectrum of Public Participation 2018 [https://organizingengagement.org/models/spectrum-of-public-participation/.

[34] Northridge ME, Metcalf SS. Enhancing implementation science by applying best principles of systems science. Health Research Policy & Systems. 2016;14:1–8. doi: 10.1186/s12961-016-0146-8 27716275 PMC5050576

[35] Metz A, Louison L, National Implementation Research N. The Hexagon: An Exploration Tool. Hexagon Discussion & Analysis Tool Instructions. National Implementation Research Network; 2019.

[36] Ugalde A, White V, Rankin NM, Paul C, Segan C, Aranda S, et al. How can hospitals change practice to better implement smoking cessation interventions? A systematic review. CA: a cancer journal for clinicians. 2022;72(3):266–86. doi: 10.3322/caac.21709 34797562

[37] Bauer T, Erdogan B, Short J. Principles of Management: FlatWorld; 2018 Novermber 2018.

[38] Vennix JAM. Group model-building: tackling messy problems. System Dynamics Review (Wiley). 1999;15(4):379–401.

[39] Hayward J, Morton S., Johnstone M., Creighton D., Allender S., Tools and analytic techniques to synthesise community knowledge in CBPR using computer-mediated participatory system modelling. Digital Medicine. 2020;3(1):1–6. doi: 10.1038/s41746-020-0230-x 32133423 PMC7031223

[40] Hovmand P, Rouwette E, Andersen D, Richardson G, Calhoun A, Rux K, et al. Scriptapedia: A Handbook of Scripts for Developing Structured Group Model Building Sessions. Social Science & Medicine—SOC SCI MED. 2011.

[41] Greene MC, Huang TTK, Giusto A, Lovero KL, Stockton MA, Shelton RC, et al. Leveraging systems science to promote the implementation and sustainability of mental health and psychosocial interventions in low- and middle-income countries. Harvard Review of Psychiatry. 2021;29(4):262–77. doi: 10.1097/HRP.0000000000000306 34241978 PMC9162158

[42] NIRN. Active Implementation Hub: Initiative Inventory: National Implementation Research Network. Frank Porter Graham Child Development Institute; 2020 [cited 2022 15 November]. https://nirn.fpg.unc.edu/resources/initiative-inventory.

[43] NIRN. Stakeholder Engagement Guidance for Implementation: National Implementation Research Network; 2020 [https://nirn.fpg.unc.edu/resources/stakeholder-engagement-guidance-implementation.

[44] Cochrane Effective Practice and Organisation of Care. Effective Practice and organisation of Care (EPOC) reviews 2023 [cited 2023 January 23rd]. https://epoc.cochrane.org/resources/epoc-resources-review-authors.

[45] Cochrane Training. Cochrane Handbook for Systematic Reviews of Interventions 2022 [cited 2023 February 2023]. https://training.cochrane.org/handbook.

