# Supplementary material for: Enhancing healthcare at home for older people in rural and regional Australia: A protocol for co-creation to design and implement system change
Source: PLoS One. 2023 Sep 8;18(9):e0290386. doi: 10.1371/journal.pone.0290386 (PMC10490867; doi:10.1371/journal.pone.0290386)
Supplement: S1 File — (DOCX) [file pone.0290386.s001.docx]

A research protocol is a complete written description of, and scientific rationale for, a research activity. In keeping with best practice, all research activity undertaken at Barwon Health should be documented in an up-to date study protocol. The Principal Investigator (PI) is responsible and accountable for designing, conducting, and monitoring the research protocol.

All research activities conducted at Barwon Health that involve human participants, their tissue or data, require ethics oversight.

The full protocol and associated documents must be submitted to REGI for ratification or approval by Barwon Health’s Human Research Ethics Committee (HREC).

# The protocol should provide sufficient detail to enable:

# Understanding of the background, rationale, objectives, study population, interventions, methods, statistical analyses;

# Ethical considerations, dissemination plans, and administration of the project;

# Replication of key aspects of project methods and conduct; and

# Appraisal of the project’s scientific and ethical rigor from ethics approval to dissemination of results.

Protocol amendments should be reported to the REGI by submitting an HREC amendment form as they occur. Information on the application process, amendments and reports can be found at <http://www.barwonhealth.org.au/research/column-1/regi>.

**PROTOCOL**

| Local solutions workshops:   \| *A DELIVER study* \| \| --- \| \|  \| |
| --- | --- | --- |
| Barwon Health Reference # 23/30  Version #1.1  Date: 16/03/2023 |
|  |
| **Author/s:**  Dr Cindy Needham, Miss Nikita Wheaton, Prof Steven Allender, Prof Anna Wong Shee, A/Prof Kevin Mc Namara, Dr Anna Ugalde, A/Prof Kate Huggins, Meg Murray, Mary Malakellis, Dr Laura Alston, Prof Anna Peeters |
| **CONFIDENTIAL**  This document is confidential and the property of Deakin University. No part of it may be transmitted, reproduced, published, or used without prior written authorization from the institution.  **STATEMENT OF COMPLIANCE**  This study will be conducted in compliance with all stipulation of this protocol, the conditions of the ethics committee approval, the NHMRC National Statement on ethical Conduct in Human Research (2007) and the ICH Guidelines for Good Clinical Practice (ICH-GCP). |

***This is a DELIVER Program Study. For more information on DELIVER please refer to Attachment – DELIVER Program manual***

**1.0 Introduction and Background Information**

In Australia and other developed countries efforts to reduce hospital admissions for older adults is a key focus to better manage the costs and resources associated(1-3). In Australia, hospital admissions to private and public hospitals that were potentially avoidable reached 748,000 (2017-2018), with 46 percent of these avoidable admissions for people aged 65 and over(4). Estimates from 2020-21 suggests potentially avoidable hospital admissions from older Australians represented 1.9 million patient days a year at a cost of $3.7 billion (2020-21)(5) with further evidence indicating these rates increase with age and remoteness(6). Most of these preventable admissions (61%) are related to chronic conditions for which could have been prevented through timely, quality primary care and prevention and management in the community, to avoid worsening of symptoms which in turn resulted in hospitalisation(4, 6-8).

World-wide, institutions are pointing to home-based care as a key strategy to improve equity of access and quality of care(9). The Australian Medical Association (AMA) estimates that by reducing avoidable hospital admissions over four years (2021-22 to 2024-25) $21.2 billion in savings could be made, one of the 11 recommendations put forward to the Australian Government emphasising the need for improvements to home-based care (5, 9). Home-based care is defined as acute or subacute care in a person’s home or health care services working with community and social services to prevent or reduce the burden of established disease through non-admitted care(10, 11). Home-based care has experienced substantial growth because of technological advancements in remote diagnostics, treatment modalities and communication platforms such as Telehealth(7). This growth has initiated research about how the delivery of home-based care improves patient outcomes, experiences of care, and decreases pressure on hospitals and overall healthcare costs (10, 11).

For many populations, home-based care efforts are unable to accommodate the heterogeneity in patient complex needs, and are heavily dependent on face-to-face interventions that place significant pressure on healthcare resources (10, 11) . The impact of this is exacerbated in marginalised populations; for example, rural, regional, and remote communities that experience increased hospitalisations and mortality (12). A review of governmental health initiatives that aim to reduce potentially avoidable hospital admissions of older adults in regional and rural areas of Australia identified a lack of programs and interventions specifically targeted to the needs of older adults in these areas; with stakeholders highlighting the importance of local specificity of health initiatives to the relevance of programs(7). In-home care was a dominant theme that emerged from stakeholder interviews, with the lack of in-home care services, long wait lists and lack of staff with appropriate multi-skilled training a barrier to older adults aging in place in regional and rural areas(7).

To strengthen health systems the World Health Organization (WHO) recommends that system science be used (13). WHO lists six building block that together form the ‘health system’ 1) health services 2) health workforce 3) health information 4) equitable access to essential medical produces, vaccines and technologies 5) health financing 6) leadership and governance(13). A systems approach considers the relationships between all the parts and players within a system (i.e., the building blocks), capturing the interplay between factors, intended and unintended consequences, effects and delays that emerge as a function of these characteristics; factors that would be missed when taking a more simplistic approach and examining only one building block within a system(14, 15). To date, a systematic approach involving other sectors (e.g., primary, allied and prehospital care) in rural and regional areas to reducing potentially avoidable hospital admissions and improving home-based care has been largely unexplored (7).

To support the implementation of interventions within the health system, earlier research in the field suggests a combined approach utilising both ‘systems science’ and ‘implementation science’ conceptual frameworks would be effective (16, 17). The Exploration, Preparation, Implementation and Sustainment (EPIS) framework(18) was developed from a review of implementation literature to inform the development of multi-component implementation strategies in the public sector service systems(19). The framework has been applied previously as a guiding framework in a large-scale collaborative multi-site project to improve care for vulnerable populations (justice-involved youth); and enhance inter-organisational relationships between juvenile justice and health (18). In this project the Exploration phase included Need Assessment in the form of a systems mapping exercise which informed the implementation strategies selected(17). The approach has also been broached in the field of Maternal and Child Health, the combination of both frameworks providing 1) through systems science the opportunities to understand the functionality of social, medical, environmental and behavioural factors of their public health programs; and 2) through implementation science the framework to translate the evidence-based interventions into practices and policies that impact health outcomes(16).

Effective implementation of evidence-based practices is critical for improving health outcomes. Poor implementation can be a major factor for why evidence-based practices do not achieve the desired impact on health outcomes. To achieve effective implementation, an understanding of the local context is needed, and evidence-based interventions or innovations need to be considered within these contexts. Applying systems thinking approaches to understanding local context and identifying evidence-based solutions is novel in health services settings. We propose that combining these two sciences to identify and implement EBP will have greater impact on health outcomes.

The use of both systems and implementation science approaches have the potential to build capacity and support health systems outside of metropolitan cities to rapidly assess, access and test the best available relevant evidence to improve service delivery, patient experience, and health outcomes. To date, there is a gap in the literature characterised by the lack of a detailed process which uses the combination of system science to understand and adapt complexity, and implementation science to optimise the delivery and adaptation of interventions. We present a protocol for working with health services and their partners to perform rapid identification, prioritisation, co-design of strategies to optimise the delivery of healthcare at home or closer to home for older people in rural and regional Australia using tools and techniques from both systems and implementation science.

**2.0 Study Objectives**

**Research questions:**

Can the use of systems and implementation science approaches be effectively used to

1) understand the factors that influence the barriers and facilitators of healthcare delivered at home or closer to home for older people living in regional and rural areas?

2) identify feasible solutions to improve service delivery, patient experience, and health outcomes of older people in rural and regional Australia?

**Aim/s:**

1. Utilise tools and techniques from systems and implementation science to perform rapid identification, prioritisation, planning and feasibility testing of local solutions to healthcare at home or closer to home with healthcare partners and stakeholder,
2. Build and evaluate a process to perform rapid identification, prioritisation, planning and testing of solutions to complex problems that can be applied in a range of settings.

*NB: herein home-based care is used to refer to healthcare delivered at home or closer to home.*

**Study objectives**

The objectives are to work with the partner health services, community members and the Primary Health Network to:

- Build “consensus maps” of the factors influencing home-based care for each local health system using Deakin’s Systems Thinking In Communities (STICKE) software.
- Analyse the unique local factors and problems that influence the effectiveness of homebased care; create causal loop diagrams; plot existing actions within this system; and identify key agents and their interactions.
- Reveal barriers to implementation, potential unintended consequences of interventions, and highest priority challenges to home-base care for older adults in rural and regional Victoria.
- Generate local solutions to optimizing home-based care and assess the feasibility and fit of these solutions guided by evidence-based practice, implementing site indicators and program indicators.
- Develop plans for the implementation of local solutions within each health service that can be presented to the organisational leadership team with the aim of seeking endorsement, resourcing and implementation support.
- Evaluate the effectiveness of utilizing the systems and implementation science approaches to identifying local solutions.

**Outcomes and significance**

The systems maps developed throughout the process will be analysed to identify key themes and factors across regional and rural areas that are influencing the effective delivery of home-based care. The process outlined in this protocol will facilitate cross-sectional input into identification and tracking of points in the system likely to be amenable to change, and with the potential to have a meaningful impact on the health of older adults in regional and rural Victoria. By utilising this participatory process each health service will generate one or more implementation plans (e.g., intervention or program) for an action that has the potential to improve or optimise the delivery of home-based care for older adults in rural and regional areas. These implementation plans can be used as a business case that can be presented to health services leaders to gain implementation support. The process has the potential to be scaled up and delivered across other health services and settings to identify effective local solutions to complex problems.

This protocol is part of a broader research project titled “Delivering Enhanced healthcare at home through optimising Virtual tools for older people in Rural and regional Australia” (DELIVER), a collaborative program with rural, regional and remote healthcare providers to identify digital and non-digital solutions to embed into home-based care to improve service delivery, patient experience, and health outcomes for the communities they serve. Health services that participate in the process may have the opportunity to work with the Centre of Digital Transformation for Health to further develop solutions that utilise digital technologies (e.g., Remote Patient Monitoring).

Supplementary 1 provides template documents (and sample documents) that will be used throughout the evaluation of the protocol implementation including:

1. The Activity Plan will be completed prior to each workshop and summarises the objectives of each workshop as outlined in the following sections.
2. The Activity Evaluation will be completed post each workshop and will summarise the outcome measures that will be collected and analysed throughout the implementation of the protocol.
3. Participant Feedback Survey at the completion of each workshop.

The following summarises the measures that will be collected throughout the implementation of the protocol that will be used to populate Activity Evaluation:

1. Participation numbers at each workshop; their role and representative organisation.
2. Combined health service causal loop diagrams (CLD) providing complex visual understanding of the system in which the reference mode is operating, the factors that are influential and how they interact.
3. Individual health service CLD, confirmed as valid and accurate from the participants point of view, providing a clear list of existing actions to address the problem, identification of priority areas for strengthening existing action or creating new action (CMC2).
4. Record of potential actions areas based on feasibility and likely impact (CMC2).
5. Record of individual Health service ‘Action Areas’ and ‘Action Ideas’ and names and contact details of people interested in taking part in Working Groups in CMC4-5 (developed in CMC 2-3).
6. Prioritised ‘Action Areas’ and ‘Action Ideas’ (analysis of survey data collected between CMC3 and CMC4).
7. Rating of prioritised Action Ideas and discussion notes from use of the Hexagon tool (CMC 4).
8. Implementation Plans, comprising the most feasible and fitting prioritised actions under each of the P-O-L-C functions (CMC 4 & 5).
9. A regional strategy document comprising implementation plan for each working group will be compiled including localised adaptations for each individual organisation.
10. A network of organisations seeking to implement similar interventions will have been formed.

As part of the broader DELIVER project additional evaluation measures will be recorded by the Evaluation Team which reflect measures outlined in the Evaluation Matrix in Supplementary File 1.

**3.0 Study Design and Methods**

This protocol describes the work before, during and after 5 interactive co-design sessions with key health service stakeholders and consumer representatives. The protocol uses many frameworks and tools to ensure system and implementation science complement each other. Table 1 describes each of the key frameworks and tools including the Exploration Preparation Implementation and Sustainment (EPIS) framework (18), methods and tools from community-based system dynamics (20) and National Implementation Science Networks (NIRN) Guidance for Engaging the Critical Perspectives (21, 22) and the Hexagon tool(23).

**Table 1. Tools and frameworks used throughout the Consensus Mapping and Co-design process**

| **Framework/Tool** | **Description** | **When is it used in the process** |
| --- | --- | --- |
| Exploration, Preparation, Implementation and Sustainment (EPIS) Framework | The Exploration, Preparation, Implementation and Sustainment (EPIS) framework (Figure 1) is a four phased guiding framework for the identification and implementation of new program or practice(23). The framework was developed through summation of the implementation literature in the public sector social and allied health in the USA and will be used to inform the integration of systems science and implementation science approaches (19). | Guiding framework for the overall process. |
| Causal Loop Diagram (CLD) | A CLD is a visual tool used in systems science for conceptualising a problem or issue and understanding the feedback loops between factors within the system(24). | Generated in workshop 1 and refined in workshop 2 and 3. |
| Hexagon Tool | The Hexagon tool is a guide for discussion and analysis for organisations to evaluate new and existing programs and practices through discussing and rating six domains which examine and rate the contextual fit and feasibility of an action, program or practice (Evidence, Usability, Supports, Capacity to Implement, Fit with Current Initiatives, Need)(23).  The ratings for each domain are between 1 (least supported by the discussion) to five (most supported). The Evidence briefs presented to stakeholders during the workshops will inform discussion for the ‘Evidence’ domain under the Program Indicators; and the CLD will be used to represent the Need indicators under the Implementing Site Indicators. Each of the questions under the remaining domains under the Program Indicators (Usability and Supports) and Implementing Site Indicators (Capacity to Implement and Fit with Current Initiatives) will be considered with input from participants(23). | Workshop 4 |
| Systems Thinking for Community Knowledge Exchange (STICKE) Software (25) | STICKE software creates a visual representation (CLD) of the group’s shared understanding of the problem. STICKE also allows practitioners to theme and group factors, track where action is already happening; where future efforts should be focused; assess feasibility and capacity to deliver actions; and track actions within the system as they evolve. | Utilised in workshops 1, 2 and 5. |
| Planning Organising Leading and Controlling (P-O-L-C) | Planning Organising Leading and Controlling (P-O-L-C) Framework which breaks down the four functional areas that need to be considered when planning to achieve an objective within an organisation(26).  The POLC frameworks supports participants to develop a 1) Plan comprising objectives and strategies, locating and confirming 2) Organisational functions (financial, operational or human), identify and engage 3) Leadership through considering leaders motivations 4) Control relating to setting performance standards and monitoring. | Workshop 4 and 5 |

**Research Team**

The DELIVER team comprise several specialists with a diversity of skills and experience who support the local solutions process at different stages. The team comprises five working groups (WG); Translation, Communication and Scaling WG (oversees communications and knowledge exchange); Rural engagement and co-design WG (supporting ongoing engagement and co-design); Local solutions WG (lead consensus mapping, co-design and prioritisation of local solutions); Program Evaluation WG (leading evaluation); and Capacity Building WG (implement new Research Translation (RTC) roles and mentoring and translation capability building across rural Victoria). The processes outlined within this protocol will be led by the Local solutions WG primarily with assistance from other WG throughout.

**Procedure**

Each CMC Workshop session will run for 3 hours with the pre- and post- work taking undertaken by the Research Team taking approximately one week each side of the workshop (Figure 1). Participants will be asked to attend up to five workshops which would involve no more than 15 hours of workshop time. In an ideal setting the entire process would be delivered over a 2-month period with workshops held in successive weeks. Workshops can however be run with a lengthier gap between, where this occurs we incorporate into the workshop a more detailed overview of the previous events to refresh participants memories. To overcome potential geographic constraints all workshops have the potential to be delivered in person and online. Where workshops are held online an additional team member is required to manage the online platform throughout the workshop (e.g., breakout rooms, slides, questions etc).

Step one of the process consists of an interactive workshop with representation by health service leaders and consumer representatives (e.g., consumer advisory groups as identified by the health service) of the target population across the region. Steps two and three will consist of two workshops which will be run with each individual health service and expand representation to include organisational leadership and service delivery staff (referred to as stakeholders from herein). In steps four and five stakeholders will form a working group to further develop a prioritized action. Where health services have prioritized a similar action, program or practice they wish to implement these health services will be brought together for workshop four and five so that they can share knowledge and collaborate throughout the process. Two workshops will be delivered for each prioritised action area (e.g., health services working groups will develop the program or practice for their organisation).

Participants within each workshop will include select people (refer to Recruitment section below) with a specific focus and responsibility for the delivery of the plans developed in preceding steps. Table 2 outlines the number of participants that are anticipated to participate in each workshop.

Table 2. Workshop participation numbers, cohort and workshop participant configuration

| Consensus Mapping & Co-design (CMC) workshop | Expected number of participants | Participant cohort | Workshop involves individual health services only or combined health service. |
| --- | --- | --- | --- |
| 1 | 10-15 *(5-10) | Key organisational leaders or stakeholders | Combined |
| 2 | 10-15 *(5-10) | Stakeholders invited to CMC1 and further key stakeholders identified based on factors captured in CMC1. | Individual |
| 3 | 10-15 *(5-10) | Stakeholders invited to CMC2 and further key stakeholders identified in CMC2. | Individual |
| 4 | 30-60 (5-10 per health service *(2-5)) | Stakeholders that have committed to a particular ‘action area’ working group in CMC 3 and/or self-nominated via survey. | Combined (health service specific working groups) |
| 5 | 30-60 (5-10 per health service *(2-5)) | Stakeholders that have committed to a particular ‘action area’ working group in CMC 3/4 and key stakeholders identified in CMC4. | Combined (health service specific working groups) |

* Smaller health services may have a smaller number of participants representative of size.

Note: The number of participants noted above is an estimate. It is possible to run workshops with fewer or more participants. Key to workshop success is not the number of participants alone but ensuring that the participants have are in a position to provide key insight into the complex problem and are in a position of influence .

**Figure 1. Flow chart of the Consensus Mapping and Co-Design Workshop Process**

**Note: timeframes may differ depending on availability of facilitators and stakeholders.**

2 weeks

2 weeks

2 weeks

2 weeks

2 weeks

**Settings and Stakeholders**

This combined Systems Thinking and Implementation Science approach involves a Consensus Mapping and Co-design (CMC) process which will be delivered over five workshops. An organisational leader from each health service will be nominated at project commencement to liaise with the Research Team throughout recruitment. CMC session participants will be adults over the age of 18 years who are organisational leaders and operational staff (clinical and non-clinical), people with lived experience, or representatives of people with lived experience of the organisational system. Within each stage, identification of participants to engage with will be guided by the National Implementation Research Network (NIRN) guidelines which highlights the importance of having the participants involved that understand the population, the problem, the community and that are empowered to engage in the decision-making (21, 22). Invitations to attend emails will be sent via email by either the health service liaison (or nominated representative) or the Research Team depending on the preference of the partner organisation. Email examples of workshop invitations can be found in Supplementary file 2.

For this study stakeholders consist of; consumers and/or consumer representatives - older people in rural Australia who may be eligible for healthcare in the home or closer to home, their careers and family; and, the staff of health services and other organisations engaged in home-based care. The setting for this study is the region of Western Victoria, Australia and will engage up to 8 health services. Identification of participants to engage within each stage will be guided by the NIRN Guidance for Engaging Critical Perspectives (21, 22).

A survey will be included in-between workshop three and four for the purpose of prioritising the actions generated in workshop three. The survey will be circulated via email using the survey platform Qualtrics to all participants engaged in the workshop to date. To reach a broader audience and people unable to attend the workshop, all participants will be invited to forward the survey onto relevant stakeholders where they see a connection between the potential action and stakeholder knowledge or experience. The purpose of the survey is prioritise the actions generated in workshop 3 and to provide participants the opportunity to elaborate on why they feel a particular action is important; and provide information on existing programs or initiatives that relate to the action. Participants will also be given the opportunity to self-nominate into a working group for a specific action area; this will assist in preparation for CMC 4 in which participants will work in action specific working groups. Supplementary file 3 and 4 provides an example of the email invitation to participants to complete the survey and an example of the survey content which includes some action examples.

# STEP ONE: Engage and develop initial casual loop diagram of identified problem

**How does this system look?**

**Objective:** to develop a consensus map of the system to illustrate all the factors that drive access to home-based care, how these factors interact and relationships of feedback. A second objective is to build the capacity of participants to use systems thinking to understand the complex system in which they operate.

**Participants and recruitment** (Pre-workshop)**:** CMC 1 will target organisations at the regional level. An organisational leader from each organisation will be nominated at project commencement to liaise with the Research Team to explore and confirm the problem statement and confirm the reference mode. The liaisons will assist with identifying, engaging and recruiting 10-15 key organizational leaders or stakeholders to participate in CMC 1. Within each stage, identification of participants to engage with will be guided by the NIRN guidelines which highlights the importance of having the participants involved that understand the population, the problem, the community and that are empowered to engage in the decision-making (21, 22) (Supplementary file 5). In CMC 1 relevant participants will ideally be organisational leaders, service delivery staff and other stakeholders (people with lived experience or their representatives) who have a local and/or a broader (i.e., state or country) understanding of the challenges facing the organisation or consumers about the problem statement.

Each participant will be invited via email to attend the series of 5 workshops, though it is expected as the participants and engagement narrows to the organisational/local level, many of these stakeholders may opt to delegate local responses to staff within each organisation. Each participant will receive a the Participant Information and Consent From (Supplementary File 9) which will provide an overview of the CMC process to improve their understanding of the systems science approach and the CMC process before commencing CMC 1. It is expected that the organisational liaison will also communicate directly with participants emphasising their role in the project and to support their involvement within the CMC process.

**Procedure:**

1. Confirm (joint) problem (Pre-workshop)

Working with these participants the research team will confirm the problem statement to underpin the regional level work and which specific aspect of home-based care is to be addressed. Described in system science as a **Reference Mode**, the problem statement describes the outcome of interest and the way in which it has changed over time. For example, reference modes might include changing rates of digital health literacy among older adults or admissions that were potentially avoidable were home-based care in effect. Semi-scripted interview questions tailored to the organization will be used in an online or in-person format to explore and confirm the Problem Statement/Reference mode for the study with the organisational leader.

1. Conduct rapid review (Pre-workshop) (RTS^[[1]](#endnote-2)^, LSWG)

Once the reference mode is set, a rapid review will be conducted for the topic area, current evidence will be identified and synthesised describing current patterns relating to the problem and the current evidence about effective solutions (27). The review will provide an overview of implementation and service outcomes that have influenced the Reference Mode (e.g., interventions that have influenced rates of potentially avoidable hospital admissions). Organisational data will also be collected to inform the problem statement and assist with evaluation (i.e., baseline measures) as well as to identify key organisation representatives and stakeholders.

The results of the rapid review and the organisational data collected (e.g., the number of older adults currently utilising home-based care programs and the number of potentially avoidable hospital admissions by older people) will be combined to form the basis of an evidence brief which will be presented to participants in the first CMC workshop. For further detail regarding the collection of organisational data please refer to Section 4.0 Data Collection. The evidence brief will be up to 20 minutes in length and tailored to ensure it is understandable to all participants.

1. Recruit and organise CMC 1

Participants for the first CMC workshop will be drawn from the same pool as the first part of step one. These participants are identified as representing either organisation leaders or service delivery staff or stakeholders (people with lived experience or their representatives) who have a regional or broader understanding of the challenges facing the organisations. They will be asked to identify others who fit this criterion and have not yet been contacted. It is anticipated that the number of participants will be between 10 and 15.

The method underpinning the workshop approach is community based participatory system dynamics (28), which comprises a suite of tightly scripted activities to identify relationships of cause and effect relating to the problem statement. The activities are facilitated by the research team and notes are taken during the process to capture the depth of discussion to inform later refinement and review (Table 3). Supplementary file 6 provides scripts to guide Workshops one to three that have been developed by the Global Centre for Preventative Health and Nutrition (GLOBE) at Deakin University which sits within the Institute for Health Transformation.

Step 2 (Rapid Review) and Step 3 (Recruitment) will likely occur concurrently.

**Table 3. Workshop format and data collection for Session one (Consensus Mapping and Co-design Workshop 1**

| Agenda item | Time (mins) | Description |
| --- | --- | --- |
| Welcome | *10* | The study lead introduces the session and the purpose of the study, welcomes people to the session and outlines the meeting structure and aims. |
| Evidence Brief | *20* | Participants are presented with an evidence brief providing the most recent evidence regarding the topic area (problem statement) and the use of potential interventions alongside data evidencing the state of play within organisations where permission has been given to share. The evidence brief also presents information on what is known about implementing interventions and/or programs within the context of the topic area. |
| Behaviours over time | *20* | Participants are presented with the reference mode comprising the change over time at the organisational and regional level of the identified problem. Participants are then asked to consider, individually, what factors are leading to, or resulting from, the patterns observed in the reference mode. Participants are asked to express these as a function of how the factor has changed (behaviour) over time. Participants then work in small groups of three to determine which of these factors should be prioritised in describing the cause and effect of the problem at the regional level. |
| Connection circle | *45* | Each of the small working groups present their priority variables in turn and these are entered into the STICKE software.^[[2]](#endnote-3)^ STICKE collects these variables and participants are then asked to identify the relationship of cause and effect between any of the variables collected. For each relationship (for e.g., digital literacy and access to healthcare advice) participants consider the most likely relationship of cause and effect, and the direction of the relationship (i.e., how does change in one variable affect change in another). The STICKE software then translates these causal relationships into an overall logic model showing each of the variables and their connections. |
| Model review | *15* | Participants review the initial model and confirm they are happy with the contents and relationships or add and edit as they see fit. |
| Session Close | *05* |  |

**Outcome:** Outcomes will align with the exploration phase within EPIS in which the service system, organisation, research group or other stakeholders consider the emergent and existing needs of the organisation and stakeholder to inform the best actions to address the problem statement(29). This step will build causal loop diagrams (CLD) based on stakeholders’ understanding of local context and the evidence of the factors driving the reference mode at the regional level. This will provide us with a complex visual understanding of the system in which the reference mode is operating, the factors that are influential and how they interact.

# STEP TWO: Capture a consensus on the problem and current actions and actors.

**Where are current actions, where are actions needed, where can we act?**

**Objective:** to engage with key stakeholders to validate the CLD built in the CMC1; consider the themes that have emerged as identified by the research team. The CLD is then used as a basis to identify any key actors and actions already active or occurring within the system; where and what action should be strengthened, reduced, or new solutions created.

**Participants and recruitment** (Pre-workshop)**:** CMC2 will target each individual health service and the same participants that attended CMC1 will be invited to the second session along with further stakeholders identified based on the factors captured in the initial CLD. It is expected that there will be between 10 and 15 participants from the health service at each CMC2 workshop (with potentially fewer from smaller health services e.g., 5-10 participants). The NIRN Guidance (30) will be used to assess and plan for the engagement of a diverse range of perspectives relevant to the topic area in consultation with organisational liaisons. Each participant will be invited via email to attend the series of workshops (Supplementary file 3 for example invitation email).

**Procedure:** Following step one the research team will review and refine the CLD based on workshop notes and identify emerging thematic areas. The first evidence brief will be revised to include the variables not covered in the first evidence brief. An inventory of relevant existing initiatives will be collected to provide a clear picture of existing programs or initiatives, polices, strategies and resource commitments in place for each health service (31). These materials will then be used as a part of step two (Table 4).

**Table 4. Workshop format and data collection for session two (Consensus Mapping and Co-design Workshop 2)**

| Agenda item | Time (mins) | Description |
| --- | --- | --- |
| Welcome | *10* | The study lead introduces the session and the purpose of the study, welcomes people to the session and outlines the meeting structure and aims. |
| Evidence Brief | *10* | Participants are presented with the revised evidence brief providing the most recent information about topic area (problem statement). The evidence brief will provide more detailed information about interventions and existing local actions relating to the CLD collected in step one. |
| Model review introduction | *25* | The process used to develop the CLD in STICKE is described to the participants. The map is presented in stages to participants by building the map up theme by theme. The meaning of the variables, direction and style of arrows representing the causal relationships are described to participants. |
| Model review | *30* | Participants are invited to review the maps of the system and place a black dot where the participant felt there was something important and a pink dot where they felt something was missing. They will be able to augment the maps and add things they felt were missing. |
| Action review introduction | *10* | Using these augmented maps participants to; identify the places on the map where existing action is happening and described this along with the part of the map the action was affecting; consider where more action was needed and describe this action and the place on the map where they felt it would act; and highlight areas of the map where they felt they had power and agency to act. These responses will be recorded on handouts and will identify existing actions, areas for more action and the potential (power to act) at the local organisation level. |
| Action ideas and prioritise | *25* | Using these maps and considering the evidence presented, participants are asked to consider actions that might be taken to positively influence the reference mode. Actions are described on the action ideas template and participants were asked to identify which parts of the map the action would impact. |
| Prioritise | *15* | Working in small groups of 2 or 3 people, participants are then asked to share their ideas with each other and prioritise these ideas in order from highest to lowest priority. They are asked to prioritise considering both the feasibility of the action and the likely impact of the action. And informed by their existing knowledge and experience. |
| Group summary to room | *20* | The small working groups created in the previous step reported their priority actions to the rest of the group and these actions were recorded and displayed at the front of the room. |
| Next steps and close | *5* | The next steps in the project are described and the meeting drawn to a close. |

**Note:** Where key stakeholders are unable to attend the CMC workshop 2 a condensed one-on-one session (online or in person) will be conducted with the stakeholder led by the organisational liaison or Research Team with feedback provided recorded and provided to the research team where relevant. It is expected that this will not exceed 3 people and each will be asked to complete the Patient Information and Consent Form (Supplementary File 9) prior to the one-on-one session.

**Outcome:** This step results in the CLD being confirmed as valid and accurate from the participants point of view, providing a clear list of existing actions to address the problem, identification of priority areas for strengthening existing action or creating new action, an understanding of where there is potential to change within the system. This workshop will be the starting point for creating a list of actions areas based on feasibility and likely impact. These actions and the actors identified as working on them/responsible for their delivery will be recorded on a register and confirmed with the assistance of organisational representatives.

**STEP THREE: Moving from action ideas to create working groups to implement actions**

**Objective:** To translate the results of the previous two sessions into firm action plans and form working groups around specific action areas

**Participants and recruitment** (Pre-workshop)**:** CMC3 will target each individual health service and stakeholder identified by the participants in step one and two who have specific roles or interests in delivery within specific health services. They will be invited to attend a workshop to develop the plans further and consider their implementation at a health service level. It is anticipated that for the six health services (to be confirmed) in the region, this session may have to be run multiple times with each to ensure each health service can participate fully. Where appropriate smaller health service may choose to combine resources for this workshop. New participants will be circulated the flyer so that they are familiarised with the process and systems dynamics techniques. This is anticipated to involve 10 to 15 stakeholders per health service (potentially less for smaller health services e.g., 5-10 participants) leading to engagement with between 100 and 150 participants across all participating health services.

**Procedure:** Following step two the research team will review and refine the consensus maps to include existing and proposed actions and actors. The research team will use workshop notes to refine the map, complete the inventory of current initiatives, and confirm stakeholders. The map will be themed by target action areas, for example, providing new digital tablets to aged people in their home and providing internet coverage might be themed under ‘Increasing access to digital tools’. These themes will define working groups in step three and the evidence briefs extended to ensure coverage of these prioritised action areas (Table 5).

**Table 5. Workshop format and data collection for session three (Consensus Mapping and Co-design Workshop 3)**

| Agenda item | Time (mins) | Description |
| --- | --- | --- |
| Welcome | *10* | The study lead introduces the session and the purpose of the study, welcomes people to the session and outlines the meeting structure and aims. |
| Evidence Brief | *10* | Participants are presented with the revised evidence brief providing the most recent information about topic area (reference mode). |
| Action ideas introduction | *25* | The process used to develop the maps and action registers in STICKE is described to the participants and the prioritised actions areas from step two introduced. |
| Working group formation – round 1 | *30* | Participants are asked to choose a working group based on the prioritised action area that best meets their current remit, interest and capacity. The working group then reviews the actions in their thematic area, prioritising these in order of likely impact and feasibility. The team then conducts its first working group meeting, collecting the names of the participants, their preferred level of commitment to the group, identifying other stakeholders that may need to be involved relevant to their local health service. Participants will also consider the actions through the lens of the WHO Health Systems Strengthening framework (13) and consider what success would look like and what resources would be required. |
| Working group formation – round 2 | *30* | The process outlined above is repeated with the opportunity for participants to contribute on a second working group. |
| Group summary to room | *20* | The small working groups created in the previous step reported their priority actions to the rest of the group and these actions are recorded and displayed at the front of the room. |
| Next steps and close | *5* | The next steps in the project are described and the meeting is drawn to a close. |

**Outcomes:** Working groups will have been formed around broad action areas and actions clearly defined and considered in terms of existing resources and support.

**STEP FOUR: Form Action Area Working Groups and explore feasibility and fit of actions and co-design an Implementation Plan**

**Objective:** to consolidate working groups across organisations based on their shared priority actions areas and confirm the feasibility and fit of the proposed actions. Co-Design an implementation plan and business case for each agreed action.

**Participants/Recruitment:** Actions defined in step three will be prioritised via a Qualtrics survey platform. The survey will be sent to the participants so far engaged in each CMC and snowballed to a wider audience of stakeholders to confirm the top three priority action areas and actions within each; and allow participants to self-nominate which working groups they would like to join. Participants will be invited to join the next workshop which will form the basis for establishing health service networks. One workshop will be held for each of the top three (or more) prioritised actions and will be attended by multiple health services who also prioritised the action; within each workshop individual health services will work together within a working group. It is anticipated that each health service will have 5-10 representatives participating in CMC 4 as part of a health service specific working group (potentially less for smaller health services e.g., 2-5 participants).

**Procedure:** Following step three the research team will review the CLD to create sub maps relevant to each of the workshop action areas. Prior to the workshop, the an inventory of actions identified by participants that relate to the action area will be checked for accuracy; and further information sought from the organisational liaison. A rapid review of the evidence will be undertaken to assess the potential effectiveness of prioritised action areas and actions in addressing the problem. Gray literature along with other relevant databases such as the Effective Practice and organisation of Care (EPOC) reviews (<https://epoc.cochrane.org/resources/epoc-resources-review-authors>) and Cochrane Handbook for Systematic Reviews of Interventions (<https://training.cochrane.org/handbook>) will also be searched for supporting evidence on best-practice.

Three or more working groups will be established based on the prioritised actions. The Hexagon discussion and analysis tool (23) questions will be used to discuss, analyse and rank the feasibility and fit of actions with participants. Each subgroup will be given a handout (or electronic copy) of the Hexagon tool (Supplementary file 7) on which they will record the numerical rating for each of the six indicators; and take notes to record their discussion and rating justification.

During the session, participants will have the opportunity to contribute to assessment and ranking of all actions prioritised for the workshop. Actions that have been positively assessed for feasibility and fit will then be operationalised using the P-O-L-C Framework(REF). Participants will be provided with a template to draft the implementation plan (Supplementary file 8).

**Outcomes:** An Implementation Plan, comprising the most feasible and fitting prioritised actions under each of the P-O-L-C functions. A regional strategy document comprising implementation plan for each working group will be compiled including localised adaptations for each individual organisation (Table 5). A network of organisations seeking to implement similar interventions will have been formed.

**Table 5. Workshop format and data collection for step four (Consensus Mapping and Co-design Workshop 4)**

| Agenda item | Time (mins) | Description |
| --- | --- | --- |
| Welcome | *10* | The study lead introduces the session and the purpose of the study, welcomes people to the session and outlines the meeting structure and aims. |
| Evidence Brief | *10* | Participants are presented with the revised evidence brief providing the most recent evidence of the effectiveness of prioritised action area and actions, the availability of evidence-based programs or guidance for best practice. |
| Hexagon tool introduction | *25* | Participants are introduced to the Hexagon discussion and analysis tool and the questions that sit under each domain, and rating sheets (23). |
| Working group formation – Hexagon Tool | *30* | Participants are asked to work in subgroups (health-service specific) with representatives from their organisation. Each subgroup will be asked to systematically work through the Hexagon tool considering the Evidence, Supports, Usability, Need, Fit and Capacity to Implement of up to three specific actions prioritised within the Action Area(23). Each indicator will then be ranked to reflect the feasibility and fit of each action considered with discussion points noted. |
| Group summary to room | *20* | Each subgroup will report their ratings from the Hexagon tool for their specific actions that were discussed and analysed, and other subgroups will be given the opportunity to provide comment and discuss. |
| Working group – Setting a Business Case: Implementation Planning | *30* | Each subgroup will be asked to draft an Implementation Plan for the most feasible and fitting action (i.e., most highly ranked) for their organisation sequentially using each of the four functions outlined in the P-O-L-C Framework (26) 1) Planning 2) Organising 3) Leading 4) Controlling. |
| Group summary to room | *20* | Each subgroup will briefly present the Implementation Plan and identify the next steps to be taken in the next 0-3 months. |
| Next steps and close | *5* | The next steps in the project are described and the meeting drawn to a close. |

# STEP 5 Implementation Planning refinement, feasibility testing and Business Case preparation.

**Objective:** To support further refinement of the Implementation Plan from step 4 at the organisational level, build capacity of organisations to use systems (including the use of STICKE software) and implementation science approaches, undertake simulated feasibility testing and prepare Business Case for presentation to organisational leaders or funding bodies.

**Participants/Recruitment:** It is anticipated that each health service will have 5-10 representatives participating in CMC 4 as part of a health service specific working group (potentially less for smaller health services e.g., 2-5 participants). Participants will be those who have committed to the particular ‘action area’ working group; further stakeholders identified in Step 4 using the Hexagon tool as a needed to ‘support’ implementation will also be invited to join the specific action area working group.

**Procedure:** Step five sets up and describes the routine operation of the working groups for the action area. The working group supported by the RT in CMC 5 will work within specific region wide action specific working groups (organisational focused) to finalise implementation plans including required resources, capacity, infrastructure, training/coaching, timelines, data collection and evaluation. Participants will be asked to further develop the four functional areas that need to be considered when planning to achieve an objective within an organisation(26) using the POLC frameworks to supports participants to develop a 1) Plan comprising objectives and strategies, locating and confirming 2) Organisational functions (financial, operational or human), identify and engage 3) Leadership through considering leaders motivations 4) Control relating to setting performance standards and monitoring.

The working group will then use STICKE to undertake a rapid feasibility test by map the actions into the CLD to understand the factors that the actions might influence, and the factors that will be included in the system should the action be implemented. Participants will be shown how to extract action area specific CLDs (utilising the function of STICKE that allows you to extract portions of the map that relate to a particular theme) to track and assess whether actions are reaching the intended targets, identify unintended consequences and adjust interventions. Using an Excel the groups establish an action register which will be updated routinely (weekly) to track process changes as part of the implementation process.

**Outcomes:** At the completion of CMC 5 Action Area working groups will have been formed and supported the development of an implementation plan for the delivery of an action within their organisation. Implementation plans will form a Business Case that includes the simulated rapid feasibility test, that can be presented to leadership to support requests for support, resourcing and future implementation.

**Consent**

The identified stakeholder will be provided with the outline of the study, the plain language statement and consent form, and the researcher’s contact details (attached to application). This will occur approximately 2-3 weeks prior to the first workshop. Stakeholders will return a consent form to the researcher to confirm their participation. If there has been no follow-up after 1.5 weeks the researcher will contact the potential participant once by phone or email. If there is no response no further follow-up will be made by the researchers. The Participant Information and Consent Form can be found in Supplementary file 9.

**4.0 Data Collection**

Organisational data collection

Organisational data will be collected by the Research Team in consultation with the Organisational Liaison from each health service. Data collected will relate directly to the reference mode which will be confirmed prior CMC 1. For example, if the reference mode is exploring why home-based care programs are not being utilised data collected will relate to the number of older adults currently utilising home-based care programs (e.g., hospital in the home) and the actual capacity of these programs (i.e., the number of older adults that could be utilising these programs). This data will be used to visually represent to workshop participants the reference mode. In CMC 1 data will be deidentified and will be presented in the form of a graph or table within the presentation. In health-service specific CMC 2 and 3 the health-serve specific data will also be presented in the form of a graph or table within the presentation.

Recording and note taking.

One or more (where there are working groups) members of the Research Team will take notes throughout each workshop to ensure all relevant information is captured. Where possible, these workshops will also be recorded, through the online platform if conducted online, or with handheld recorders if in person. Consent for the recording will be sought from participants through the Patient Information and Consent Form. Participants will also be reminded of this at the beginning of the workshops. Recordings will be transcribed, removing any identifying information. The original recording will be stored on the Deakin University secure research data store platform Syncplicity, and destroyed once transcription is complete.

Data from the workshop participants

The data collected on the participants at the CMC sessions will include their role in the organisation (e.g Health Service manager) and how long (years, months) they have been in the position and whether they live locally.

The resultant CLDs from the CMC workshops will be approved by all workshop participants. These CLD’s will form part of analysis as will the outputs from the feasibility analysis (workshop 4) and implementation plans (workshop 4 and 5).

Survey data collected ranking actions and action areas will be exported into an Excel spreadsheet and the number of times each action was prioritised as one, two or three will be tallied. Participants that have self-nominated into a particular action area working group will be recorded by the Research Team and invited to attend following workshops where that action is the focus.

**5.0 Data Management**

Data from the workshop participants

The CMC workshops will generate CLDs (outlined in section 3) which will form part of the analysis. Participant demographics (name, organisation, role, email) will be gathered at the start of the workshop (completed on personal devices), or as a paper version (Supplementary file 10).

Workshop participants

Workshop participants may withdraw during the workshop process, or not attend subsequent workshops. The impact of this will be dependent on that participant’s role, however the process will be able to continue with a core number of participants.

Data storage

Where hard copy data is collected it will stored in non-identifiable form, in a locked storage office at Deakin University, for a minimum of seven years after completion of the DELIVER Program. Electronic information (i.e. workshop reports, map files etc) will also be non-identifiable, and will be stored on secure, Deakin University Syncplicity drives for a minimum period of seven years after completion of the DELIVER Program.

Accessing data

The investigators named on this ethics application will have access to the data and produce outputs as approved by the DELIVER Translation, Communications and Scale up Team.

Following the appropriate retention period, hard copy data will be destroyed, electronic data will be archived in compliance with Deakin University's data archiving policies.

**6.0 Data Analysis**

**Analysis of quantitative data**

Sample size

This study aiming to recruit up to 20 stakeholders per health service partner. Analysis of the data will not be reliant on obtaining a sample of high statistical power.

Analysis methods

A number of specialised systems analysis methods may be applied to these data across the group of communities. Specific analyses will include:

- Network analysis of systems maps
  - Network analysis can be applied to one or more of the systems maps that have arisen from community consultation in participating communities to identify structurally important features within the systems map. This can be used to help identify points of similarity or difference/uniqueness between communities, or between comparable maps informed by literature or expert opinion.
- Implementation and action tracking over time
  - Systems maps may be aligned with information about the status and progress of actions over time ay be compare across health services and will create a real-time resource for monitoring the implementation of actions from a systems-thinking perspective
- Case study/reflective qualitative analyses
  - Case studies afford an opportunity to qualitatively analyse insights from the range of potential data sources. These analyses will focus on reflective, practitioner focussed, applied research which may present studies on successful implementation of systems practice led by the councils, tracing insights from community consultation, through to the actions informed by those insights (tracked through implementation records) and concordant implications for community outcomes and implementation practise (through outcome and systems practise evaluations).

Data identifiability and community participation

The resulting prioritised action areas and action ideas will be shared with all workshop participant via email following completion of the workshops.

**7.0 Results, Outcomes and Future Plans**

Plan for return of results of research to participants

The research may be reported in peer-reviewed research articles, theses, written reports and/or conference presentations. Only aggregated and de-identified data will be presented from the evaluation survey data.

Individual research results will not be returned to participants, but workshop participants will be provided with an overall summary report of the workshop outcomes, that does not include identifiable information at the cessation of the program.

There are no risks to participants in sharing the summary results as these are presented in a non-identifiable form discussing general, non-sensitive or personal issues about ways to improve the community food environment.

Plans for dissemination and publication of project outcomes

Project outcomes will be disseminated through conference abstracts, peer-review publications and presentations at appropriate community and health service forums, as well as academic conferences and symposiums. Lay summaries will be developed for the DELIVER website and other digital formats.

**8.0 Budget and Funding**

The study is funded by a Commonwealth funded MRFF Rapid Applied Research Translation Grant (RARUR000072). DELIVER funding supports one employee 0.5FTE to plan and deliver each of the workshops with the assistance of CI Allender. A budget of approximately $3000 per workshop is allocated for workshop delivery support costs such as room and equipment hire, catering, materials, facilitator travel, accommodation, and meals.

Additional members of the DELIVER team will support evidence compilation for workshop evidence briefs. DELIVER will have the potential to support and evaluate the development and implementation of actions that fit within the scope of the project (e.g. interventions that include a digital home-based care component).

Uncategorized References

1. Huntley AL, Chalder M, Shaw ARG, Hollingworth W, Metcalfe C, Benger JR, et al. A systematic review to identify and assess the effectiveness of alternatives for people over the age of 65 who are at risk of potentially avoidable hospital admission. BMJ open. 2017;7(7):e016236.

2. D'Souza S, Guptha S. Preventing admission of older people to hospital. BMJ (Clinical research ed). 2013;346:f3186.

3. van den Broek S, Heiwegen N, Verhofstad M, Akkermans R, van Westerop L, Schoon Y, et al. Preventable emergency admissions of older adults: an observational mixed-method study of rates, associative factors and underlying causes in two Dutch hospitals. BMJ open. 2020;10(11):e040431.

4. Australian Institue of Health and Welfare. Disparities in potentially preventable hospitalisations across Australia, 2012-13 to 2017-18. Canberra: AIHW; 2020.

5. AMA. Putting health care back into aged care. Online: file:///C:/Users/ncindy/Downloads/Putting%20health%20care%20back%20into%20aged%20care.pdf; 2021.

6. Falster MJ, L.,. A guide to the potentially preventable hospitalisations indicator in Australia. . Sydney: University of New South Wales in consultation with Australian Commission on Safety and Quality in Health Care and Australian Institute of Health and Welfare; 2017.

7. McManamny TE, Boyd L, Sheen J, Lowthian JA. Health initiatives to reduce the potentially preventable hospitalisation of older people in rural and regional Australia. Health Promotion Journal of Australia. 2022;33(3):553-65.

8. Clinical Epidemiology and Health Service Evaluation Unit. Potentially preventable hospitalisations: a review of the literature and Australian policies. Melbourne, Victoria: Royal Melbourne Hospital; 2009.

9. Commonwealth of Australia. Royal Commission into Aged Care quality and Safety. Prime Minister and Cabinet; 2021.

10. Victorian Clinical Council. Home-based care becoming the new norm. 2020.

11. Healthcare Improvement Scotland. Hospital at Home: Guiding principles for service development. 2020.

12. AIHW. Rural and Remote Health Online: <https://www.aihw.gov.au/reports/rural-remote-australians/rural-and-remote-health>: Australian Government; 2022 [

13. WHO. Strenthening health systems to improve health outcomes: WHO's framework for action. Geneva: World Health Organisation,; 2007.

14. Allender S, Owen B, Kuhlberg J, Lowe J, Nagorcka-Smith P, Whelan J, et al. A Community Based Systems Diagram of Obesity Causes. PLoS ONE. 2015;10(7):1-12.

15. Mabry PL, Olster DH, Morgan GD, Abrams DB. Interdisciplinarity and systems science to improve population health: a view from the NIH Office of Behavioral and Social Sciences Research. American Journal of Preventive Medicine. 2008;35(2(Suppl. 1)):S211-S24.

16. Kroelinger C, Rankin K, Chambers D, Diez Roux A, Hughes K, Grigorescu V. Using the Principles of Complex Systems Thinking and Implementation Science to Enhance Maternal and Child Health Program Planning and Delivery. Maternal & Child Health Journal. 2014;18(7):1560-4.

17. Knight DK, Belenko S, Wiley T, Robertson AA, Arrigona N, Dennis M, et al. Juvenile Justice-Translational Research on Interventions for Adolescents in the Legal System (JJ-TRIALS): a cluster randomized trial targeting system-wide improvement in substance use services. Implementation Science. 2016:1-18.

18. Becan JE, Bartkowski JP, Knight DK, Wiley TRA, DiClemente R, Ducharme L, et al. A model for rigorously applying the Exploration, Preparation, Implementation, Sustainment (EPIS) framework in the design and measurement of a large scale collaborative multi-site study. Health & justice. 2018;6(1):9.

19. Aarons GA, Hurlburt M, Horwitz SM. Advancing a conceptual model of evidence-based practice implementation in public service sectors. Administration and Policy in Mental Health and Mental Health Services Research. 2011;38(1):4-23.

20. Hovmand P, Rouwette E, Andersen D, Richardson G, Calhoun A, Rux K, et al. Scriptapedia: A Handbook of Scripts for Developing Structured Group Model Building Sessions. Social Science & Medicine - SOC SCI MED. 2011.

21. National Implementation Research Network. Guidance for Engaging Critical Perspectives 2023 [

22. International Association for Public Participation. Spectrum of Public Participation 2018 [Available from: <https://organizingengagement.org/models/spectrum-of-public-participation/>.

23. Metz A, Louison L, National Implementation Research N. The Hexagon: An Exploration Tool. Hexagon Discussion & Analysis Tool Instructions. National Implementation Research Network; 2019.

24. Vennix JAM. Group model-building: tackling messy problems. System Dynamics Review (Wiley). 1999;15(4):379-401.

25. Hayward J, Morton, S., Johnstone, M., Creighton, D., Allender, S.,. Tools and analytic techniques to synthesise community knowledge in CBPR using computer-mediated participatory system modelling. Digital Medicine. 2020;3(1):1-6.

26. Bauer T, Erdogan B, Short J. Principles of Management: FlatWorld; 2018 Novermber 2018.

27. Greene MC, Huang TTK, Giusto A, Lovero KL, Stockton MA, Shelton RC, et al. Leveraging systems science to promote the implementation and sustainability of mental health and psychosocial interventions in low- and middle-income countries. Harvard Review of Psychiatry. 2021;29(4):262-77.

28. Hovmand PS. Community based system dynamics. New York, NY: Springer Science + Business Media; 2014.

29. Moullin J, Dickson K, Stadnick N, Rabin B, Aarons G. Systematic review of the Exploration, Preparation, Implementation, Sustainment (EPIS) framework. Implementation Science. 2019;14(1):1-16.

30. NIRN. Stakeholder Engagement Guidance for Implementation: National Implementation Research Network; 2020 [Available from: <https://nirn.fpg.unc.edu/resources/stakeholder-engagement-guidance-implementation>.

31. NIRN. Active Implementation Hub: Initiative Inventory: National Implementation Research Network. Frank Porter Graham Child Development Institute; 2020 [cited 2022 15 November]. Available from: <https://nirn.fpg.unc.edu/resources/initiative-inventory>.

1. RTS: Rapid Translation Specialist [↑](#endnote-ref-2)
2. Hayward J, Morton S, Johnstone M, Creighton D, Allender S. Tools and analytic techniques to synthesise community knowledge in CBPR using computer-mediated participatory system modelling. NPJ digital medicine. 2020;3(1):1-6.

   Supplementary links:

   [Hexagon tool NIRN](chrome-extension://efaidnbmnnnibpcajpcglclefindmkaj/https:/nirn.fpg.unc.edu/sites/nirn.fpg.unc.edu/files/imce/documents/NIRN%20Hexagon%20Discussion%20Analysis%20Tool_September2020_1.pdf)

   [Guidance for Engaging Critical Perspectives](chrome-extension://efaidnbmnnnibpcajpcglclefindmkaj/https:/nirn.fpg.unc.edu/sites/nirn.fpg.unc.edu/files/resources/Guidance%20for%20Engaging%20Critical%20Perspectives%20January%202023.pdf)

   [Initiative Inventory](https://nirn.fpg.unc.edu/resources/initiative-inventory) [↑](#endnote-ref-3)
